# Rift Valley fever virus is able to cross the human blood–brain barrier *in vitro* by direct infection with no deleterious effects

**DOI:** 10.1128/jvi.01267-24

**Published:** 2024-09-30

**Authors:** Jordan Quellec, Camille Piro-Megy, Marion Cannac, Sébastien Nisole, Florent H. Marty, Fabien Gosselet, Fumitaka Shimizu, Takashi Kanda, Catherine Cêtre-Sossah, Sara Salinas

**Affiliations:** 1 ASTRE, CIRAD, INRAE, University of Montpellier, Montpellier, France; 2 PCCEI, University of Montpellier, INSERM, Etablissement Français du Sang, Montpellier, France; 3 IRIM, CNRS UMR9004, University of Montpellier, Montpellier, France; 4 Blood Brain Barrier Laboratory, Faculty of Science Jean Perrin, Artois University, Lens, France; 5 Department of Neurology and Clinical Neuroscience, Yamaguchi University Graduate School of Medicine, Ube, Japan; University of Michigan Medical School, Ann Arbor, Michigan, USA

**Keywords:** Rift Valley fever virus, blood brain barrier, neuroinvasion, immune response, endothelium integrity

## Abstract

Rift Valley fever (RVF) is a zoonotic arboviral disease that causes recurrent epidemics in Africa that may trigger fatal neurological disorders. However, the mechanisms of neuroinvasion by which the RVF virus (RVFV) reaches the human central nervous system (CNS) remain poorly characterized. In particular, it is not clear how RVFV is able to cross the human blood–brain barrier (hBBB), which is a neurovascular endothelium that protects the brain by regulating brain and blood exchanges. To explore these mechanisms, we used an *in vitro* hBBB model to mimic *in vivo* hBBB selectiveness and apicobasal polarity. Our results highlight the ability of RVFV to cross the hBBB by direct infection in a non-structural protein S (NSs)-independent but strain-dependent manner, leading to astrocyte and pericyte infections. Interestingly, RVFV infection did not induce hBBB disruption and was associated with progressive elimination of infected cells with no impairment of the tight junction protein scaffold and barrier function. Our work also shows that NSs, a well described RVFV virulence factor, limited the establishment of the hBBB-induced innate immune response and subsequent lymphocyte recruitment. These results provide *in vitro* confirmation of the ability of RVFV to reach human CNS by direct infection of the hBBB without altering its barrier function, and provide new directions to explore human RVFV neurovirulence and neuroinvasion mechanisms.

**IMPORTANCE:**

The RVF virus (RVFV) is capable of infecting humans and inducing severe and fatal neurological disorders. Neuropathogenesis and human central nervous system (CNS) invasion mechanisms of RVFV are still unknown, with only historical studies of autopsy data from fatal human cases in the 1980s and exploration studies in rodent models. One of the gaps in understanding RVFV human pathogenesis is how RVFV is able to cross the blood–brain barrier (BBB) in order to reach the human CNS. For the first time, we show that RVFV is able to directly infect cells of the human BBB *in vitro* to release viral particles into the human CNS, a well-characterized neuroinvasion mechanism of pathogens. Furthermore, we demonstrate strain-dependent variability of this neuroinvasion mechanism, identifying possible viral properties that could be explored to prevent neurological disorders during RVFV outbreaks.

## INTRODUCTION

The emergence of zoonotic viral infections in recent decades is linked to increased human activities, global warming, and environmental changes (1, 2). It is therefore crucial to better understand emerging vector-borne viral diseases by improving our knowledge of arthropod-borne viruses (arboviruses) and host interactions (1). Neurological disorders triggered by neurotropic viruses have become a serious burden and highlight the need for both specific prevention strategies and efficient treatment (3–5). One of these viral diseases is Rift Valley fever (RVF), first described in Kenya in 1930, that is caused by the arbovirus Rift Valley fever virus (RVFV) (6). RVFV is a tripartite and negative single-stranded RNA virus of the *Bunyaviricetes* class and belongs to *Phlebovirus riftense* species (7–9). RVFV has high dissemination capacities, with reports of alarming, re-occurring, and ongoing epidemics in endemic countries in Africa, the South West Indian Ocean region, and the Arabian peninsula (9–12). RVFV epidemics are a serious threat to both animal and human health (8, 9, 13). However, despite the increased risk of emergence of RVF at a global scale, there are still no commercially available vaccines or specific antiviral treatment for RVFV infection in humans (14). Consequently, RVFV is rated as Class A bioterrorism agent by US Centers for Diseases Control and Prevention (CDC), and the World Health Organization (WHO) classified RVFV on the Blueprint list of “Pathogens prioritization” as a prototype pathogen with pandemic potential that represents a public health emergency of international concern.

Human pathogenesis of RVFV is still poorly understood, with numerous questions remaining unanswered. RVFV is transmitted from infected livestock to humans either by direct contact through aerosolized particles or *via* mosquito bites (9, 15, 16). The human mortality rate from RVFV infection ranges from 0.5% to 2% but has reached higher rates in some epidemics (9, 17, 18). Clinical manifestations of RVF in humans range from flu-like symptoms to less common but severe clinical manifestations associated with higher mortality (17–19). These severe manifestations take four main forms: acute hepatitis, hemorrhagic fever, ocular diseases, and neurological disorders (17, 18). Poorly characterized in humans, neurological disorders have a variable prevalence (<1% to 21%) and are mainly characterized by a delayed meningoencephalitis occurring from 1 to 4 weeks post-infection, with up to 50% mortality and potentially definitive neurological sequalae (17–19). Associated with severe neurological symptoms (e.g., convulsion, hallucination or coma), human brain damage is poorly described in the literature, but has been reported to involve tissue necrosis with lymphocyte and macrophage infiltration, lymphocytic pleocytosis in the cerebrospinal fluid and perivascular cuffing (16–18, 20–22).

RVFV neuropathogenesis has been studied using several rodent and non-human primate (NHP) models, but the question of how RVFV is able to reach the central nervous system (CNS) remains unclear (17, 23–26). In rodents, it was recently demonstrated that after infection by aerosolization of viral particles, RVFV was able to reach the CNS by infection of the olfactory epithelium, across the cribriform plate that protects olfactory receptor neurons (27). CNS infection in rodents is characterized by (i) massive viral replication, (ii) late breakdown of the blood–brain barrier (BBB), and (iii) late immune response associated with infiltration of leukocytes. Immune response and leukocytes are needed for viral clearance of RVFV, but may worsen damage to brain tissue associated with severe encephalitis (28–33). In NHPs showing clinical signs of encephalitis after RVFV infection, infection of neurons in the brain was also confirmed by histological examinations of brain tissue, although neuroinvasion mechanisms were not explored (23). Similar to animal models, the neuroinvasion mechanisms used by RVFV to reach the human CNS remain unknown (25, 26, 34).

Given the crucial roles the CNS plays in global homeostasis, it is immuno-privileged and isolated from physiological changes, toxins, and pathogens by the presence of several cell barriers that regulate exchanges between the CNS and other compartments of the body (35–37). The neurovascular unit (NVU) is one of these barriers that regulate exchanges between the blood and the CNS (37). The NVU is a complex organization of different cell types: supporting cells (astrocytes, pericytes, and microglia) help and regulate the neurovascular endothelium, more commonly named the blood–brain barrier (BBB) (36, 37). Human BBB (hBBB) is composed of specific endothelial cells, the human brain microvascular endothelial cells (hBMECs), forming a poorly permeable vascular endothelium with specific characteristics: (i) an apicobasal polarity associated with specific cell transporters able to regulate exchanges between the bloodstream and the brain and (ii) highly expressed specific tight junction (TJ) proteins between cells to ensure that the endothelium is poorly permeable and that paracellular spaces are sealed (35–38). One of these TJ proteins is the cytoplasmic TJ-associated zonula occludens-1 (ZO-1), whose role is to organize transmembrane TJ proteins in an intercellular scaffold (35). Disruption of ZO-1 leads to breakdown of the BBB and neurological disorders (35). Pathogens may use different routes to reach the CNS and cross the BBB: (i) the axonal transport following infection of synapses or axons from the peripheral nerve or from the olfactory tract, or (ii) the hematogenous routes (39, 40). This last mechanism includes different ways of crossing the BBB from the blood compartment and of releasing viral particles into the CNS, these ways include direct BMEC infection, para- or transcellular diffusion, or Trojan horse mechanism by infection of immune cells that are naturally able to transmigrate through the BBB (39, 40).

Mechanisms used by RVFV during viremia to cross the human BBB have not been characterized to date (26). To explore RVFV neuroinvasion mechanisms, we used an *in vitro* human BBB model composed of human endothelial cells (hEC) differentiated into human brain-like endothelial cells (hBLEC) after co-culture with human brain pericytes, thus mimicking blood (apical) and brain (basal) compartments, as well as *in vivo* hBBB selectiveness and specific markers (41–45). Using this model, we first characterized hBBB permissiveness and integrity following RVFV infection. We also examined the role in RVFV neuroinvasion of RVFV non-structural protein NSs, a major virulence factor implicated in numerous viral mechanisms, and in particular, inhibition of cell immune response (46, 47). Finally, we explored the immune response induced by RVFV-infected hBBB and the role of astrocytes in hBBB disruption.

## RESULTS

### RVFV differentially infects human cell types that make up the hBBB

To characterize RVFV neuroinvasion mechanisms, we decided to explore separately the permissiveness of each of the cell types composing a human BBB model previously described in literature (41, 44, 45). As a first approach, we explored the permissiveness of human endothelial cells (hEC) not yet differentiated into specific endothelial cells of the hBBB (hBLEC), and human brain pericytes.

**Fig 1:**
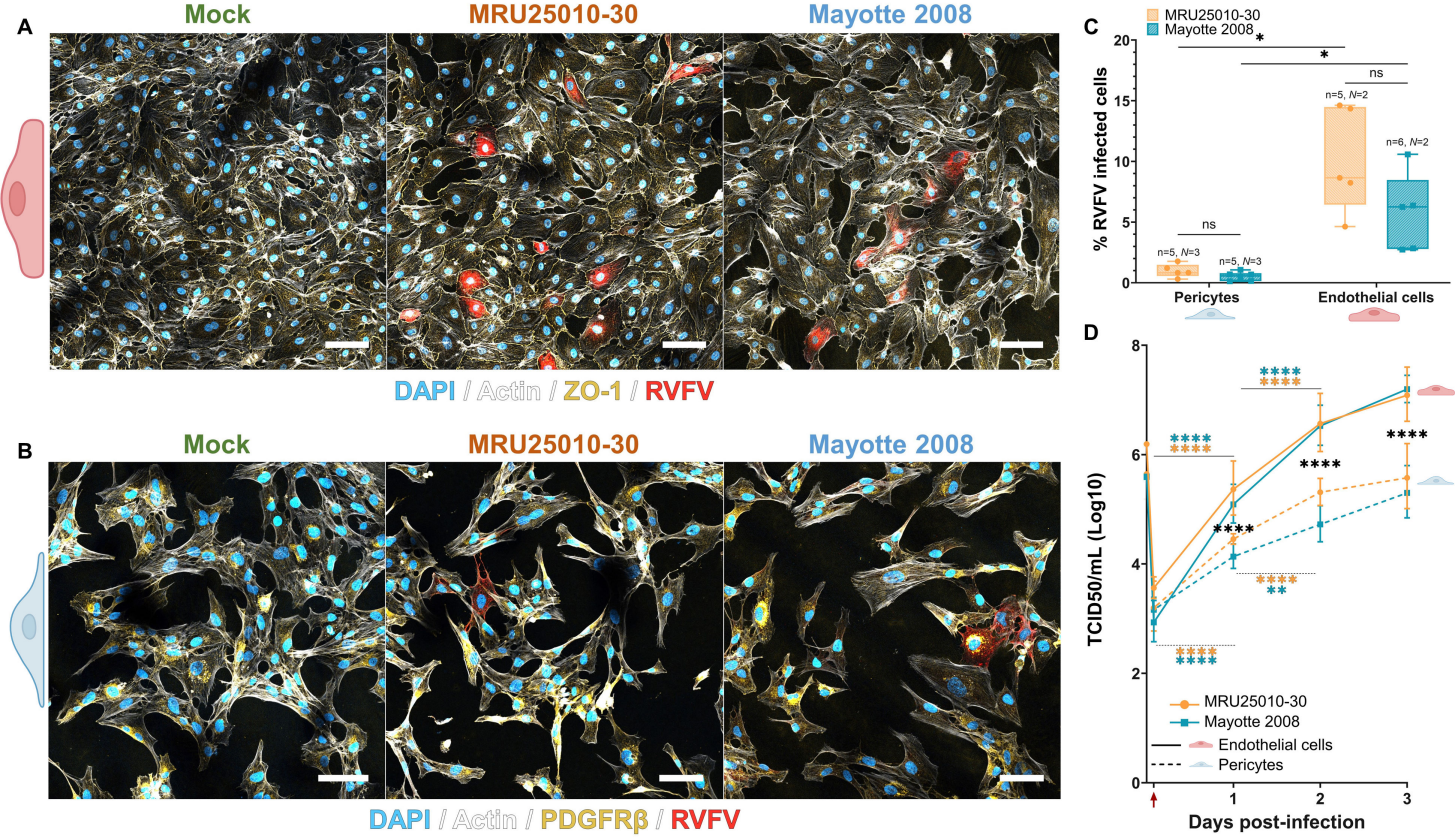
RVFV infects and replicates in the endothelial cells and pericytes that make up the human blood brain barrier (BBB) differently. Human primary endothelial cells (hEC) and human primary pericytes were infected by two distinct RVFV strains (MRU25010-30 and Mayotte 2008) or cell medium only (mock) at a multiplicity of infection (MOI) of 1. At 24 hpi, the cells were fixed and stained with DAPI (blue, nucleus), an actin probe (white, cytoskeleton), a RVFV-specific anti-N antibody (red, RVFV), and cell-specific markers (yellow) depending on the type of cell: (A) hEC (ZO-1) and (B) pericytes (PDGFRβ). Images were analyzed using confocal microscopy and are representative of each merged staining per condition. Scale bar, 20 µm. (C) From confocal microscopy images representative of a minimum of two independent experiments, the percentage of RVFV-infected cells for each cell type was analyzed by counting labeled infected cells compared with the total number of cells at 48 hpi (counting ≥500 cells per replicate, generalized linear model, *P* < 0.05). (D) Supernatants of mock- and RVFV-infected cells (solid line for hEC, dashed line for pericytes) were harvested at different time points post-infection and titrated using the TCDI50 method (red arrow: removal of inoculum and washing). Results are representative of a minimum of two independent experiments (*n* = 6/*N* = 2 for endothelial cells and *n* = 7, 12, 12, 6/*N* = 3, 4, 4, 2 for pericytes) and are expressed as geometric mean ± 95% confidence interval (CI). Linear model showed significance between titers of each cell type at the different time points (black, strain-independent) or between titers of two different time points for each strain and each cell type (blue for Mayotte 2008, orange for MRU25010-30) (*P < 0.05, **P < 0.01, ****P < 0.0001).

Each cell type was infected at a multiplicity of infection (MOI) of 1 using two distinct RVFV strains (MRU25010-30 and Mayotte 2008). Viral infection was first assessed using an immunostaining approach: we observed the ability of the two RVFV strains to infect at 1 day post-infection (dpi) hEC (labeled specifically with the ZO-1 TJ marker) (Fig. 1A) and pericytes (labeled specifically with the PDGFRβ marker) (Fig. 1B). At 2 dpi, a significant difference in the quantification of infected cells was observed between the two cell types but not between the two RVFV strains for each cell type (Fig. 1C). RVFV infection was estimated at 2 dpi at 0.8% ± 0.4% and 7.9% ± 5% for pericytes and hEC, respectively, with similar rates for MRU25010-30 and Mayotte 2008. This difference was confirmed when RVFV titers were determined using the TCID50 method at several time points post-infection for both RVFV strains and for each cell type (Fig. 1D). In hEC, MRU25010-30 and Mayotte 2008 RVFV strains displayed a significant replication up to 2 dpi. In accordance with the percentage of infected cells, there was no significant difference in titers between the two strains. In pericytes, MRU25010-30 and Mayotte 2008 displayed a significant replication up to 2 dpi, but, unlike in hEC, the two strains had different viral titers at 2 dpi (LM, *P* < 0.05). Independently of the RVFV strain, there was a potent and significant difference between titers of each cell type at each time point post-infection (*i.e*., from 1 to 3dpi).

These results revealed permissiveness of human endothelial cells and brain pericytes but significant variability in the cell type-dependent infection rate. Indeed, RVFV infected hEC more efficiently than pericytes. Moreover, RVFV showed strain-dependent variations in replication in pericytes but not in hEC.

### RVFV is able to cross the human BBB by direct infection

**Fig 2:**
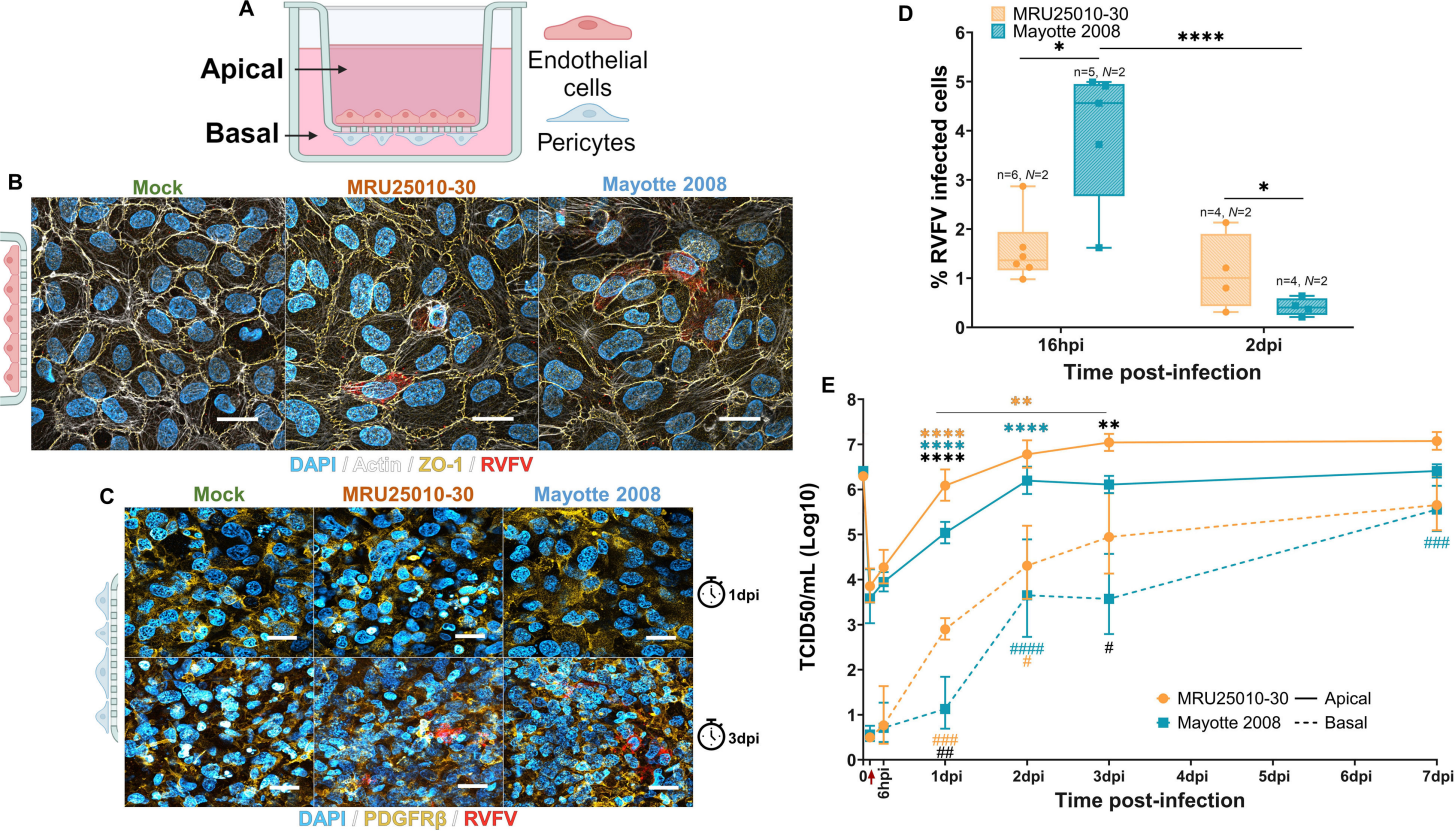
RVFV can cross the human BBB by direct infection of human brain-like endothelial cells (hBLECs). (A) *In vitro* model of human BBB consisting of human endothelial cells plated on the apical side of transwells and differentiated in hBLEC by co-incubation with human brain pericytes plated on the basal side of the transwell. BBB was infected on the apical side with two RVFV strains (MRU25010-30 and Mayotte 2008) or medium only (mock) at a MOI of 1. After infection, the cells were fixed and stained with DAPI (blue, nucleus), actin probe (white, cytoskeleton), RVFV-specific anti-N antibody (red, RVFV), and cell markers (yellow) depending on the cell type: (B) hBLEC (ZO-1) at 1 dpi (day post-infection) and (C) pericytes (PDGFRβ) at 1 dpi (top panels) and 3 dpi (bottom panels). Transwells were analyzed by confocal microscopy and are representative of each merged labeling for each condition. Scale bar, 10 µm. (D) Percentage of RVFV-infected hBLECs were analyzed by counting labeled infected cells compared with the total number of cells at 16 hpi (hours post-infection) and 2 dpi (counting ≥1,000 cells, generalized linear model (GLM): *P < 0.05, ****P < 0.0001). (E) Supernatants of RVFV- and mock-infected BBB (solid line: apical supernatants, dashed line: basal supernatants) were harvested at different time points post-infection and titrated using the TCDI50 method (red arrow: removal of inoculum and washing). Results are representative of at least two independent experiments (*n* = 6, 6, 9, 6, 9, 9/*N* = 2, 2, 3, 2, 3, 3) and are expressed as geometric mean ± 95% confidence interval (CI). GLM for each compartment independently shows significant difference in the apical compartment (*) or in the basal compartment (#) between titers of each strain at the different time points (black) or between titers of a time point with the previous time points for each strain (blue for Mayotte 2008, orange for MRU25010-30: *P < 0.05, **P < 0.01, ***P < 0.001, ****P < 0.0001).

Despite the description of neurological disorders related to RVFV human infections, the neuroinvasion mechanisms used by RVFV to reach the brain are still unknown. In order to study these mechanisms as well as the impact of the cellular differentiation on the RVFV infection, we differentiated the hECs described before into BBB-specific endothelial cells by co-cultivation with human brain pericytes. The hEC derived from CD34+ hematopoietic cells were then plated on the apical side of transwells and co-incubated with human brain pericytes that were plated on the other side of the transwell (basal side) 6 days before infection (Fig. 2A), allowing further differentiation of the hEC in human brain-like endothelial cells (hBLECs). This *in vitro* human BBB model mimics the blood compartment (apical) and the brain parenchyma compartment (basal) of brain blood micro-vessels and displays similar characteristics than *in vivo* hBBB (low permeability, specific markers, channels, tight junction proteins, and selectiveness) (43–45). This *in vitro* model was extensively used in order to characterize CNS invasion mechanisms by several viruses (48–52).

The *in vitro* hBBB model was infected at a MOI of 1 on the apical side to mimic viremia during infection with both MRU25010-30 and Mayotte 2008 RVFV strains. The susceptibility of the two cell types that comprise mature hBBB (i.e., hBLEC and pericytes) was determined by immunostaining. Using this approach, we showed that each strain was able to infect hBLEC at 24 hpi (Fig. 2B), but pericytes only at 3 dpi (Fig. 2C), suggesting that infection of pericytes occurred following infection and crossing of the hBLEC monolayer by RVFV strains. Determination of the percentage of infected hBLEC showed a much lower percentage of infected cells than in hEC in monoculture conditions (Fig. 2D). At early time post-infection (*i.e*., 16hpi), 4% ± 1.5% cells were infected by Mayotte 2008, and 1.6% ± 0.7% cells were infected by MRU25010-30. This infection rate remained stable for MRU25010-30 at 2 dpi but was lower for Mayotte 2008 (0.4% ± 0.3%).

Despite the lower percentage of RVFV-infected hBLEC compared with hEC, we showed an efficient replication of RVFV in hBLEC. RVFV titers, determined by the TCID50 method at different time points post-infection in both strains and in both compartments, reached similar titers as described in other types of cells known to be permissive to RVFV infection, such as astrocytes and liver cell lines (Fig. 2E) (47, 53). The RVFV strain MRU25010-30 showed a potent and significant replication at the apical side the first day post-infection and kept on replicating up to 3 dpi. The RVFV strain Mayotte 2008 showed a weaker but nevertheless significant replication up to 2 dpi. There was a significant difference between the titers of the two strains at 1 and 3 dpi, but this difference was no longer significant at 7 dpi (Fig. 2E). Significantly, the RVFV strain MRU25010-30 crossed from apical to basal compartments during the first 24 hpi, whereas the RVFV strain Mayotte 2008 crossed from the apical to the basal side later, only detected at 2 dpi and kept on increasing until 7 dpi, in line with its lower apical replication. In the basal compartment, there was a significant difference between the two RVFV strain titers between 1 and 3 dpi, but this difference was no longer significant at 7dpi (Fig. 2E).

These results highlighted the ability of RVFV to infect hBLEC and cross the BBB, illustrated by efficient RVFV replication, consistent with a mechanism of neuroinvasion through direct infection of the hBBB. Independently of the RVFV strain, there was a significant correlation between apical and basal titers (Spearman test, r = 0.841, *P* < 0.0001, *n* = 90/*N* = 3) as well as between the increase in the apical titers and in the basal titers up to 3 dpi (Pearson test, r = 0.707, *P* < 0.05, *n* = 8/*N* = 3). These correlations suggest a crucial role of RVFV replication for neuroinvasion. However, viral kinetics during hBBB infection and neuroinvasion *in vitro* seemed to be strain-dependent.

### Maintenance of hBBB integrity during RVFV infection

**Fig 3:**
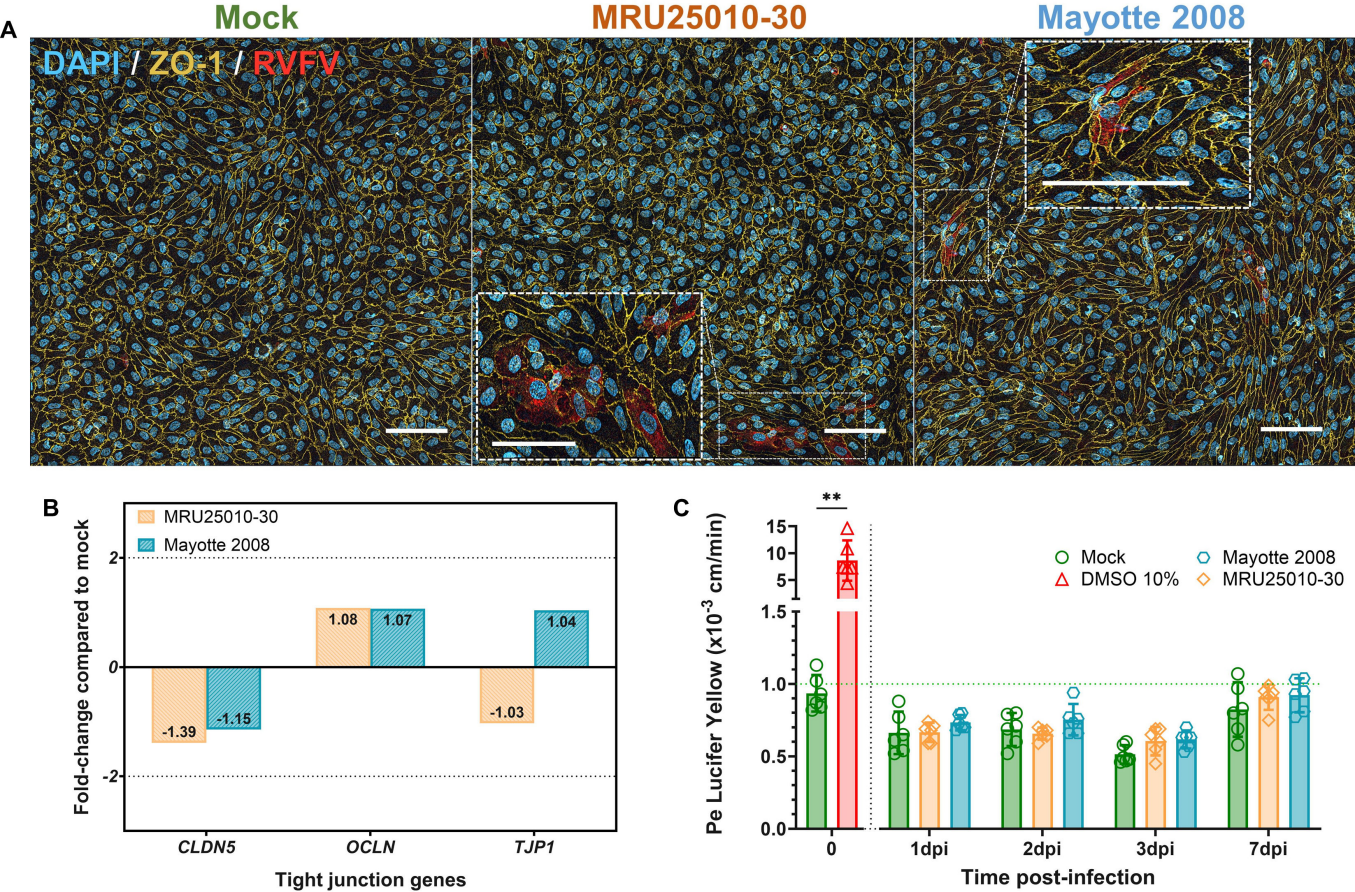
RVFV infection of human BBB did not impair tight junction protein scaffolds and barrier integrity. Human BLECs were infected on the apical side of the transwell with two distinct strains of RVFV (MRU25010-30 and Mayotte 2008) or medium only (mock), MOI 1. (A) At 2 days post-infection, the cells were fixed and stained with DAPI (blue, nucleus), RVFV-specific anti-N antibody (red, RVFV) and anti-tight junction (TJ) protein ZO-1 (yellow). Transwells were analyzed by confocal microscopy, and images are representative of each labeling merged for each condition. Dashed squares correspond to a zoom on the region of interest. Scale bar, 100 µm. (B) Expression of TJ proteins in RVFV-infected hBLEC (MRU25010-30 and Mayotte 2008, MOI 1) at 48 hpi was assayed by measuring gene mRNA expression with RT-qPCR using the fold-change method standardized with the housekeeping gene *HPRT1*. Genes were considered as upregulated with a fold-change >2 or downregulated with a fold-change <-2 (one-way ANOVA on Delta Ct values with Tukey correction, *n* = 6/*N* = 2). (C) The permeability coefficient (Pe) was measured using the Lucifer Yellow transport assay for mock and RVFV-infected BBB at different time points during infection. Treatment with 10% DMSO for 30 min corresponds to the positive control of this assay (impaired integrity) and Pe <1 × 10^−3^ cm/min (green dashed line) corresponds to an impermeable/tight BBB. Results are expressed as mean ± 95% confidence interval (CI) and a Mann–Whitney test between DMSO control and mock at T0 validated the assay (*n* = 6/*N* = 2, **P < 0.01). Generalized linear model (GLM) was used to test significance between Pe of BBB infected by each strain and compared with mock controls at the different time points (*n* = 6/*N* = 2).

After assessing hBBB permissiveness to RVFV infection, we explored the impact of RVFV infection on hBBB integrity and barrier function, since RVFV is a well-characterized lytic virus. To this end, we infected the human *in vitro* BBB model at a MOI of 1 with the two RVFV strains MRU25010-30 and Mayotte 2008, and at 2 dpi, performed immunostaining of TJ protein ZO-1 on large fields of the hBBB in RVFV and mock-infection conditions (Fig. 3A). First, we observed the maintenance of tight junction protein scaffolds, more specifically through the location of the TJ protein ZO-1 in the cell membrane, which is widely considered to be a crucial determinant of BBB maintenance (35). Independently of the RVFV strain, this qualitative approach demonstrated that RVFV infection of hBLEC did not alter ZO-1 expression or location compared to mock conditions. More precisely, RVFV-infected cells expressed ZO-1 at cell junctions, suggesting that infected cells were still associated with intact TJ proteins at this stage and that RVFV infection of hBLEC did not lead to hBBB disruption. Furthermore, we quantified the expression of mRNA encoding several TJ proteins in hBLEC infected with RVFV for 48 h by RT-qPCR (Fig. 3B). Consistent with our previous observations, we did not detect any upregulation (fold-change >2) or downregulation (fold-change <-2) in RVFV-infected hBLEC of the three TJ protein transcripts tested: Claudin-5 (*CLDN5*), Occludin (*OCLN*), and ZO-1 (*TJP1*). This result suggests that RVFV infection had no effect on TJ gene expression. To further confirm this observation, we quantified transcellular hBBB permeability at different time points during infection. To this end, we used a Lucifer Yellow assay to determine a coefficient of permeability (Pe) (Fig. 3C). As described in the literature, a Pe <1 × 10^−3^ cm/min corresponds to an impermeable or “tight” BBB (43, 54). Compared with mock-treated hBBB, each strain did not induce significant impairment of hBBB permeability at each time point analyzed, not even at 7dpi.

Taken together, these results suggest that RVFV is able to cross the human BBB by direct hBLEC infection without disrupting TJ scaffolds and with no deleterious effects on hBBB barrier integrity.

### Infection of hBBB by RVFV leads to the elimination of infected cells from the hBLEC monolayer

**Fig 4:**
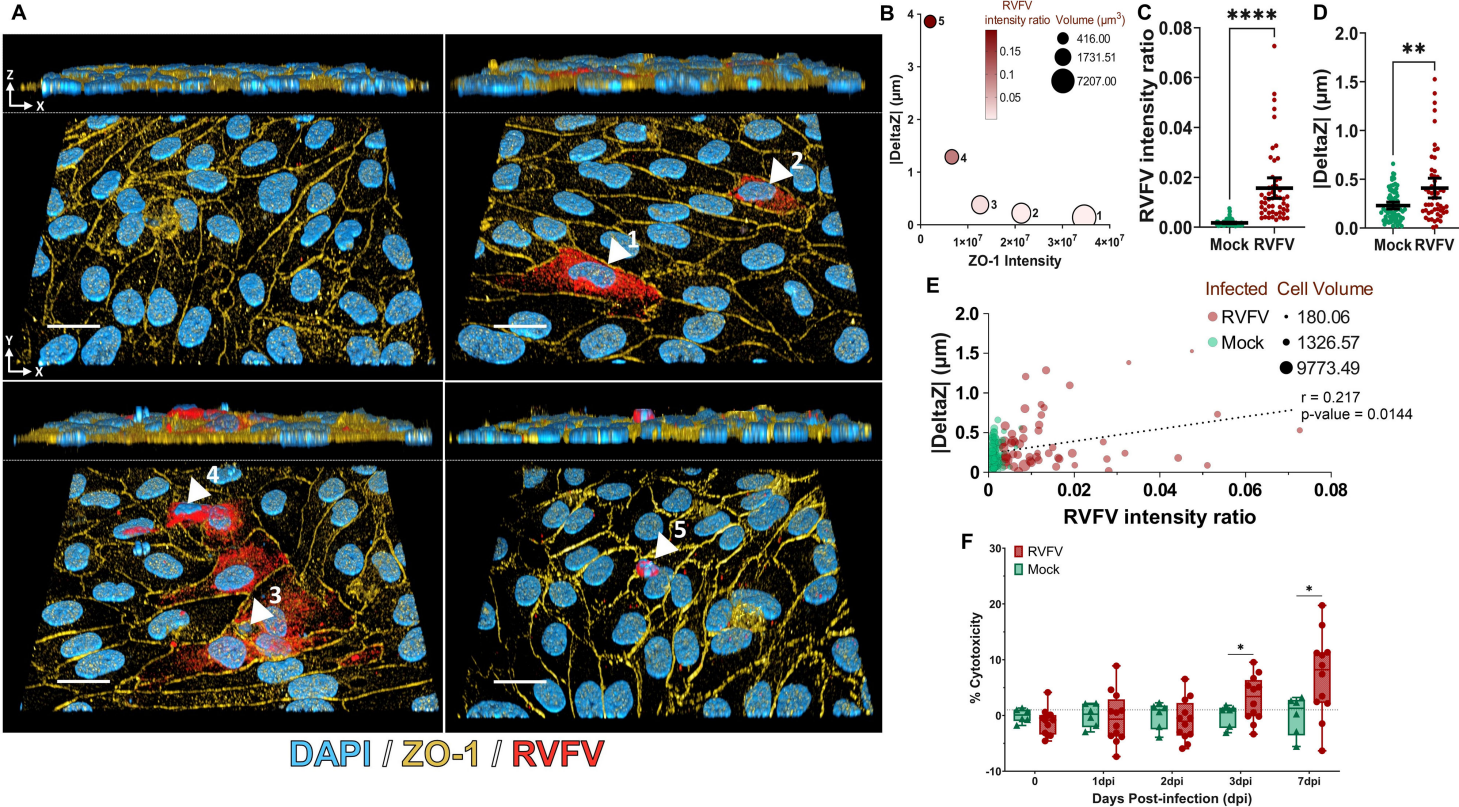
RVFV-infected hBLECs were gradually eliminated from the endothelial monolayer. In order to explore impact of RVFV on hBLEC viability and elimination, the BBB model was infected on the apical side with RVFV or medium only (mock), MOI 1. (A) At 16 hpi and 1 dpi, cells were fixed and stained with DAPI (blue, nucleus), RVFV-specific anti-N antibody (red, RVFV), anti-GAPDH (not represented), and anti-tight junction (TJ) protein antibody (yellow, ZO-1). Images were acquired using confocal microscopy and are representative of each staining merged for each condition (3D Viewer, ImageJ software). For each image, the top panel corresponds to the view on the Z-axis, the associated bottom panel to the view on the Y-axis. White arrows indicate different states of infected cells numbered by their infectious state according to the RVFV intensity ratio (mean of RVFV labelling fluorescence/volume): early (1) to late infected cells (5). Scale bar, 20 µm. (B) For the cells numbered 1 to 5, RVFV intensity ratio, cell volume (µm^3^), absolute values of cell height compared with the mean of uninfected cell height of the layer (|DeltaZ|) and fluorescence intensity corresponding to ZO-1 labeling were obtained using Imaris software. For each condition, RVFV- and mock-infected cells analyzed by confocal microscopy and Imaris software (*n* = 52/*N* = 4 and *n* = 74*/N* = 2, respectively), (C) RVFV intensity ratios were determined for each cell. Ratios are represented as mean ± 95% confidence interval (CI) (Mann–Whitney, *P*-value: ****P < 0.0001). (D) |DeltaZ| were determined for each cell and are represented as mean ± 95% CI (CI) (Mann-Whitney, **P < 0.01). (E) For each cell, |DeltaZ|, cell volume (µm), and RVFV intensity ratio were represented, and correlation between |DeltaZ| and RVFV intensity ratio values were confirmed by Spearman test (r = 0.217, *n* = 126*/N* = 4, *P* < 0.05). (F) To explore cytotoxicity of hBLEC during RVFV infection, LDH activity was measured in the apical supernatant after several days post-infection (dpi) and is expressed as percentage of cytotoxicity. Significance between RVFV and mock control were analyzed by two-way ANOVA model (*n* = 6*/N* = 2 for mock-infected cells and *n* = 12*/N* = 2 for RVFV-infected cells, *P < 0.05)

Because of the low percentage of RVFV-infected hBLEC compared with hEC and the maintenance of hBBB integrity despite RVFV infection, we decided to explore the fate of infected cells. Using immunostaining, we observed gradual elimination of RVFV-infected cells from the hBLEC monolayer (Fig. 4A). Infected hBLEC became progressively rounder and higher on the Z-axis compared with adjacent uninfected cells. We observed that higher cells were smaller, and their expression of ZO-1 decreased (Fig. 4B). Nevertheless, despite infection, cells kept expressing ZO-1, corroborating our previous results. We confirmed this observation by 3D quantitative microscopy after labeling cells with anti-GAPDH (cell cytoplasm staining), anti-RVFV, and anti-ZO1 antibodies. Indeed, by measuring the height on the Z-axis (|DeltaZ|), RVFV fluorescence (RVFV intensity ratio, Fig. 4C) and cell volume for RVFV- and mock-infected cells, we first showed that RVFV-infected cells were significantly higher than mock-infected cells (Fig. 4D). We highlighted a significant and positive correlation between cell height and RVFV fluorescence (Spearman test, r = 0.217, *n* = 126/*N* = 4, *P* = 0.0144) (Fig. 4E). Our data confirmed a significant and negative correlation between RVFV fluorescence and cell volume (Spearman test, r = −0.234, *n* = 126 *N* = 4, *P* < 0.01), as well as between the volume of infected cells and their height (Spearman test, r = −0.321, *n* = 52/*N* = 4, *P* < 0.01). To assess whether RVFV hBLEC infection led to cell mortality, we measured LDH activity, a well-known cytoplasmic enzyme marker of cytotoxicity, in apical supernatants at several time points post-infection. Using this approach, we showed that the percentage of cytotoxicity became significantly higher from 3 dpi in RVFV-infected conditions compared with mock-infected hBLEC (Fig. 4F).

Although these results suggest that hBLEC RVFV infection leads to moderate cell mortality, it seems that the hBBB maintains its integrity during RVFV infection by gradually eliminating infected cells from the monolayer without disrupting cell junctions, thereby corroborating maintenance of the endothelium during infection.

### Cell-dependent and polarized modulation of the RVFV-induced immune response by hBBB

**Fig 5:**
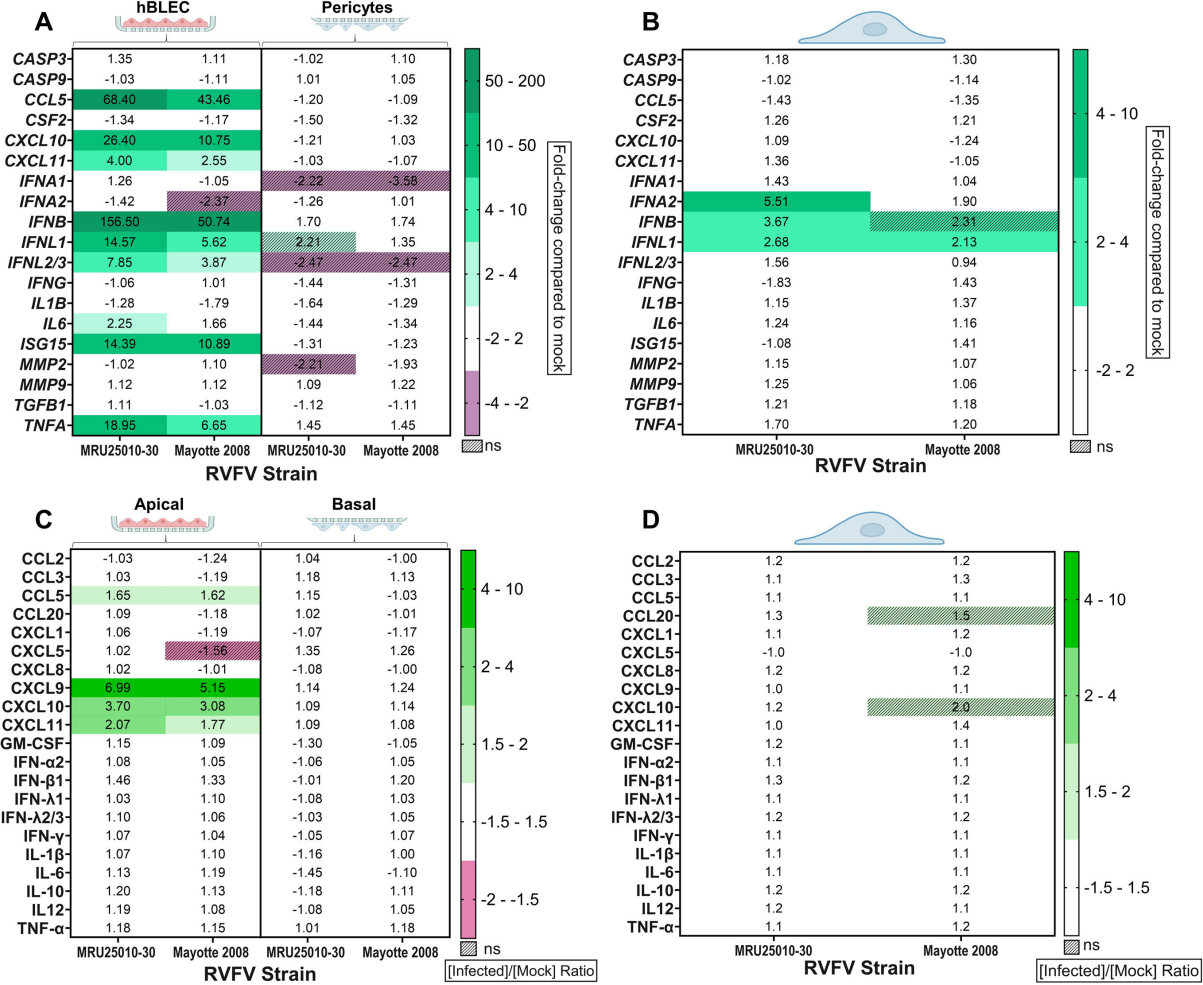
hBLEC but not pericytes induced a moderate type I interferon response after RVFV infection. Immune response induced by RVFV-infected human BBB (MRU25010-30 and Mayotte 2008, MOI 1, hBLEC, and pericytes on transwells) at 48 h post-infection (hpi) (A) or immune response induced by RVFV-infected pericytes in monoculture (MRU25010-30 and Mayotte 2008, MOI 1) after 24 hpi (B) was assayed by measuring gene mRNA expression with RT-qPCR using the fold-change method standardized on the housekeeping gene *HPRT1* (upregulated in green; downregulated in purple). All upregulated or downregulated genes compared with mock expression have a *P* < 0.05 (For each gene, one-way ANOVA or Kruskal–Wallis on Delta Ct values for each compartment independently, *n* = 6/*N* = 2) or are hatched when not-significant). Cytokines secreted in RVFV-infected BBB supernatants (MRU25010-30 and Mayotte 2008, MOI 1, apical and basal) (C) or in RVFV-infected pericyte supernatants in monoculture (MRU25010-30 and Mayotte 2008, MOI 1) (D) at 48 hpi were measured using FACS multiplex assays and compared to mock concentrations. Results are expressed as protein levels with the ratio of mean protein concentration in infected conditions to that in mock conditions (upregulated in green; downregulated in red). Modifications of protein expression represented on the heatmap have a *P* < 0.05 or are hatched when not-significant (for each protein, one-way ANOVA or Kruskal–Wallis on protein concentration values for each compartment independently, *n* = 6/*N* = 2).

As mentioned above, we observed that RVFV infection at the apical side reached a plateau at 3 dpi, suggesting control of viral replication by the hBBB. To test this hypothesis, we explored the hBBB innate immune response induced by RVFV infection with MRU25010-30 and Mayotte 2008 at MOI of 1. We measured mRNA expression of different genes implicated in the modulation of the innate immune response and cell viability in hBLEC and human pericytes. At 48 hpi, the mRNA extracted from each type of cell was quantified by RT-qPCR (Fig. 5A). Only hBLEC mRNA showed moderate upregulation of several genes implicated in the innate immune response: type I interferon (*IFNB, ISG15*) and inflammatory response (*CCL5, CXCL10, CXCL11,* and *TNFA*). However, we did not observe any upregulation of *IFNA* or gene involved in the type II interferon response (*IFNG*). Confirming published work on BBB antiviral activity (55), we observed an upregulation of mRNA encoding type III interferons (*IFNL1, IFNL2/3*) for both strains. Consistent with our previous results, there was no change in expression of apoptosis-associated genes (*CASP3*, *CASP9*) or of genes implicated in the disruption of the BBB structure, such as matrix metalloproteases (MMPs: *MMP2/MMP9*). Moreover, we detected no significant changes in the expression of selected genes in pericytes (at the basal side of the hBBB) at 2 dpi. There was a significant difference in gene expression between the two RVFV strains in hBLEC: only MRU25010-30 induced upregulation of *IL6* mRNA expression, and a significantly greater increase in *IFNL1* and *CXCL10* expression (*P* < 0.05). To confirm the absence of innate immune response in pericytes upon RVFV infection of the hBBB, we repeated the experiment at 1 dpi on monocultures (Fig. 5B). Only weak upregulation of *IFNL1* mRNA expression was observed after infection of pericytes by both RVFV strains. In addition, *IFNA2* and *IFNB* were upregulated only when cells were infected by the RVFV MRU25010-30 strain. Finally, there was no upregulation of *ISG15*, suggesting that IFN response was not triggered.

Because of the role of RVFV NSs protein in the inhibition of mRNA nuclear export, we also explored the immune response induced by RVFV infection at the protein level. We observed significant upregulation in the apical compartment of some cytokines and chemokines, including proteins upregulated at the mRNA level, e.g., CCL5, CXCL9, CXCL10, and CXCL11 (Fig. 5C). However, at 2 dpi, we did not detect any significant upregulation of IFN-β1 or IFN-λ1 at the protein level, despite the upregulation at the mRNA level. Nevertheless, the upregulation of CXCL10 (at the protein level) or *ISG15* mRNA, both of which are induced by type I interferon, indirectly demonstrated the efficient establishment of the IFN response upon infection. Interestingly, there was no significant strain-dependent difference in the upregulation at the protein level, suggesting that despite a different level of mRNA upregulation, immune response was induced at the same level in the two RVFV strains. We did not detect any change at the protein level in the basal compartment, thereby confirming the absence of innate immune response elicited by pericytes at the basal side at 48 hpi. We further investigated this absence in monoculture pericytes infected with RVFV and observed no significant modulation of cytokine and chemokine expression (Fig. 5D).

These results demonstrated a moderately polarized strain-dependent innate immune response on the apical side of the hBBB, mediated solely by hBLEC.

### Neuroinvasion of RVFV is NSs-independent, although NSs is required for the control of RVFV-induced hBBB immune response

**Fig 6:**
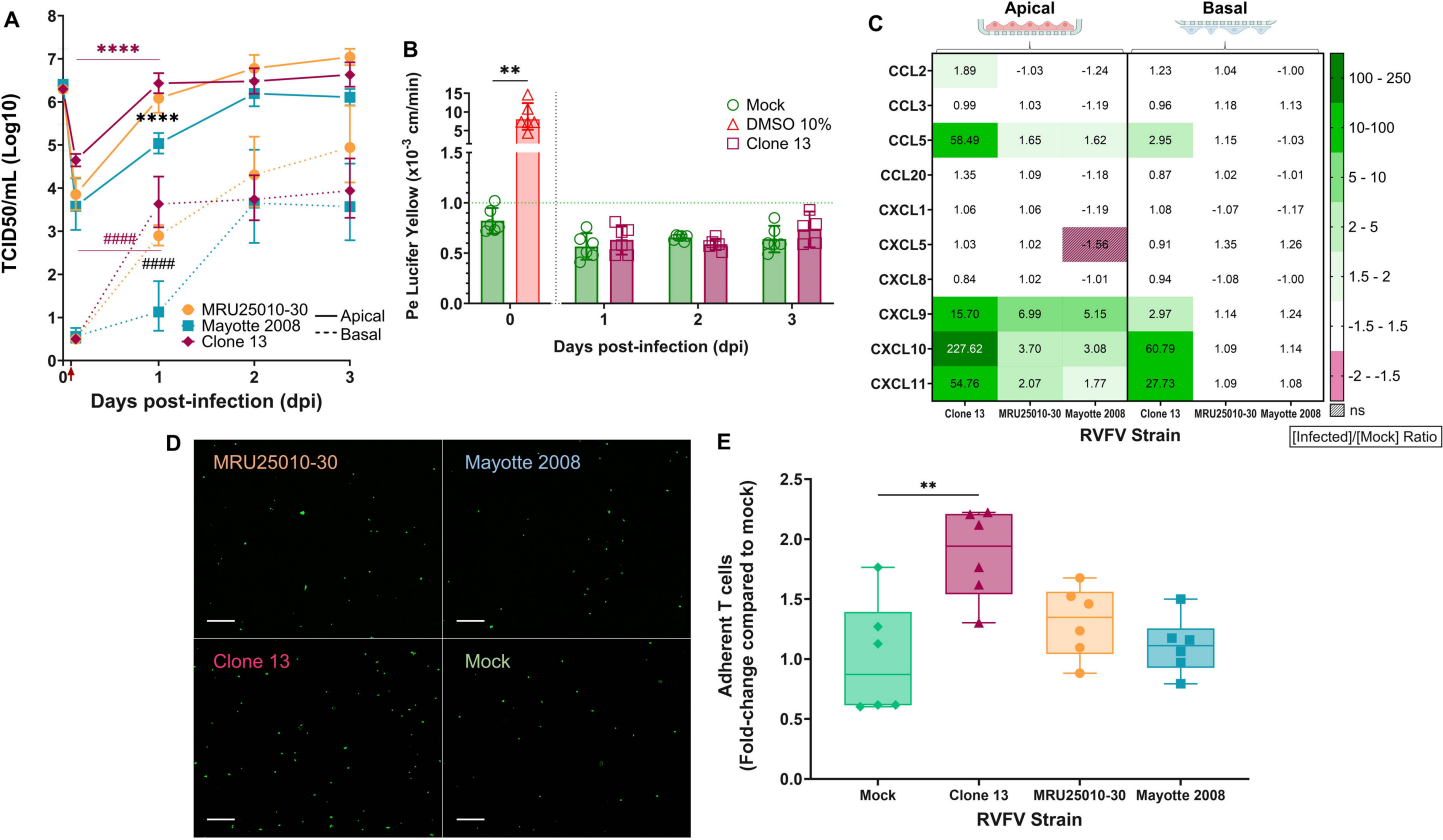
The lack of virulence factor NSs did not impair RVFV neuroinvasion but induced a strong immune response by blood–brain barrier (BBB). In order to explore the ability of Clone 13, a naturally NSs-deleted RVFV strain, to reach the brain (basal) from the blood (apical) compartment by crossing BBB, we infected the BBB model on the apical side with three RVFV strains (MRU25010-30, Mayotte 2008, and Clone 13) or medium only (mock), MOI 1. (A) Supernatants of mock- and RVFV-infected BBB (solid line: apical supernatants, dashed line: basal supernatants) were harvested at different time points post-infection and titrated using the TCDI50 method (red arrow: removal of inoculum and washing). Results are expressed as geometric mean ± 95% confidence interval (CI) (*n* = 6,9,6,9/*N* = 2,3,2,3 for MRU25010-30 and Mayotte 2008, also presented in Fig. 2E, and *n* = 6/*N* = 2 for Clone 13). Generalized linear model (GLM) for each compartment independently showed the significance of others strains in the apical (*) or basal compartment (#) compared with titers of Clone 13 at different time points (black) or between Clone 13 titers a time point with the previous time point (magenta, ****P < 0.0001). (B) The permeability coefficient (Pe) was measured using the Lucifer Yellow transport assay for mock and Clone 13-infected BBB at different time points post-infection. Results are expressed as mean ± 95% confidence interval (CI). Pe <1 × 10^−3^ cm/min (green dashed line) corresponds to an impermeable/tight BBB. Treatment with 10% DMSO for 30 min corresponds to the positive control of this assay (impaired integrity, Mann–Whitney test, *n* = 6/*N* = 2, **P < 0.01). GLM test significance between Pe of BBB infected by each strain compared with the mock control at the different time points (*n* = 6/*N* = 2, with the exception of Clone 13 at 3 dpi *n* = 5/*N* = 2). (C) Cytokines secreted in RVFV-infected BBB supernatant (apical and basal) at 48 h post-infection (hpi) were measured using FACS multiplex assays and compared with mock. Results are expressed as protein levels by a ratio of mean protein concentration in infected conditions to that in mock condition (upregulation in green, downregulation in red). Modifications of protein expression represented on the heatmap have a *P* < 0.05 or are hatched when non-significant (for each protein, one-way ANOVA or Kruskal–Wallis on protein concentration values for each compartment independently, *n* = 6/*N* = 2 for infected conditions and *n* = 12/*N* = 4 for mock). Data presented for MRU25010-30 and Mayotte 2008 are also presented in Fig. 5C. (D) At 48 hpi, apical supernatants of RVFV-infected BBB were harvested and co-incubated overnight with human primary T cells. T cells were then CFSE-labeled (green), incubated in the same infected BBB at the apical side and analyzed by acquisition of panoramas of 20 fields with tile modules. Scale bar, 100 µm. (E) Adherent CFSE-labeled cells were counted for each condition and fold-change of adherent T cell compared with mock was determined for each condition by dividing the number of cells by the mean of number of cells in the mock condition (one-way ANOVA, *n* = 6/*N* = 2, **P < 0.01)

To explore the potential role of the major RVFV virulence factor NSs in neuroinvasion mechanisms, and notably in hBBB infection and crossing, we used the Clone 13 RVFV strain, a naturally NSs-deleted strain (56). We infected the *in vitro* human hBBB model on the apical side with a MOI of 1 and quantified the viral titers at several post-infection time points (Fig. 6A). Interestingly, Clone 13 was able to infect hBLEC, had a significant replication, and crossed the hBLEC monolayer in the first 24 hpi, as illustrated by a significant increase in viral titers in the apical and basal compartments. Unlike other strains, replication of Clone 13 appeared to be controlled after 1 dpi as there was no significant difference between titers at day 1, 2, or 3 post-infection in both basal and apical compartments. Despite the lack of a major virulence factor, Clone 13 replicated more efficiently than Mayotte 2008 and as efficiently as the most virulent strain, MRU25010-30. Indeed, at 1 dpi, there was a significant difference between viral titers of Clone 13 and Mayotte 2008, but no difference between Clone 13 and MRU25010-30. Moreover, in the same way as with MRU25010-30 and Mayotte 2008 RVFV strains, infection of hBLECs with Clone 13 did not disrupt hBBB integrity (Fig. 6B). To understand why Clone 13 replication reached a plateau as early as 1 dpi, we evaluated hBBB innate immune response at 2 dpi at the protein level (Fig. 6C). We showed that Clone 13 induced a stronger inflammation than MRU25010-30 and Mayotte 2008, potentially inhibiting Clone 13 replication. Only Clone 13 induced upregulation of CCL2 expression in the apical compartment, as well as stronger and significant upregulation of CCL5, CXCL9, CXCL10, and CXCL11 than MRU25010-30 and Mayotte 2008 (Kruskal–Wallis test, *P* < 0.05). Moreover, only Clone 13 induced upregulation of these cytokines in the basal compartment. Thus, it seems that the lack of NSs induced a stronger innate immune response, and unlike virulent viruses, in both compartments. Furthermore, we explored the role of this innate immune response in leukocyte recruitment at the hBBB, which is an important feature of neuroinflammation during RVFV encephalitis (29). To do so, we co-incubated harvested apical supernatant of 2 dpi RVFV- or mock-infected hBBB with human primary lymphocytes overnight, and then co-incubated the lymphocytes with the corresponding RVFV- or mock-infected hBBB. After 45 min of co-incubation, the cells were washed, and the number of adherent lymphocytes was quantified (Fig. 6D and E). Using this experimental approach, we were able to show that RVFV infection increased lymphocyte adhesion to the hBBB by a factor of 2, but only in the case of the NSs-deleted strain Clone 13.

Taken together, these results suggest that RVFV NSs virulence factor is not crucial for neuroinvasion by direct infection but produces a stronger innate immune response, limiting RVFV replication and potentially enhancing neuroinflammation (cytokine/chemokine expression and leukocyte recruitment). Thus, by limiting the establishment of the innate immune response, NSs might be necessary for efficient replication of RVFV in the brain after crossing the hBBB.

### Infection of hBBB triggers human astrocyte infection but without disrupting BBB integrity

**Fig 7:**
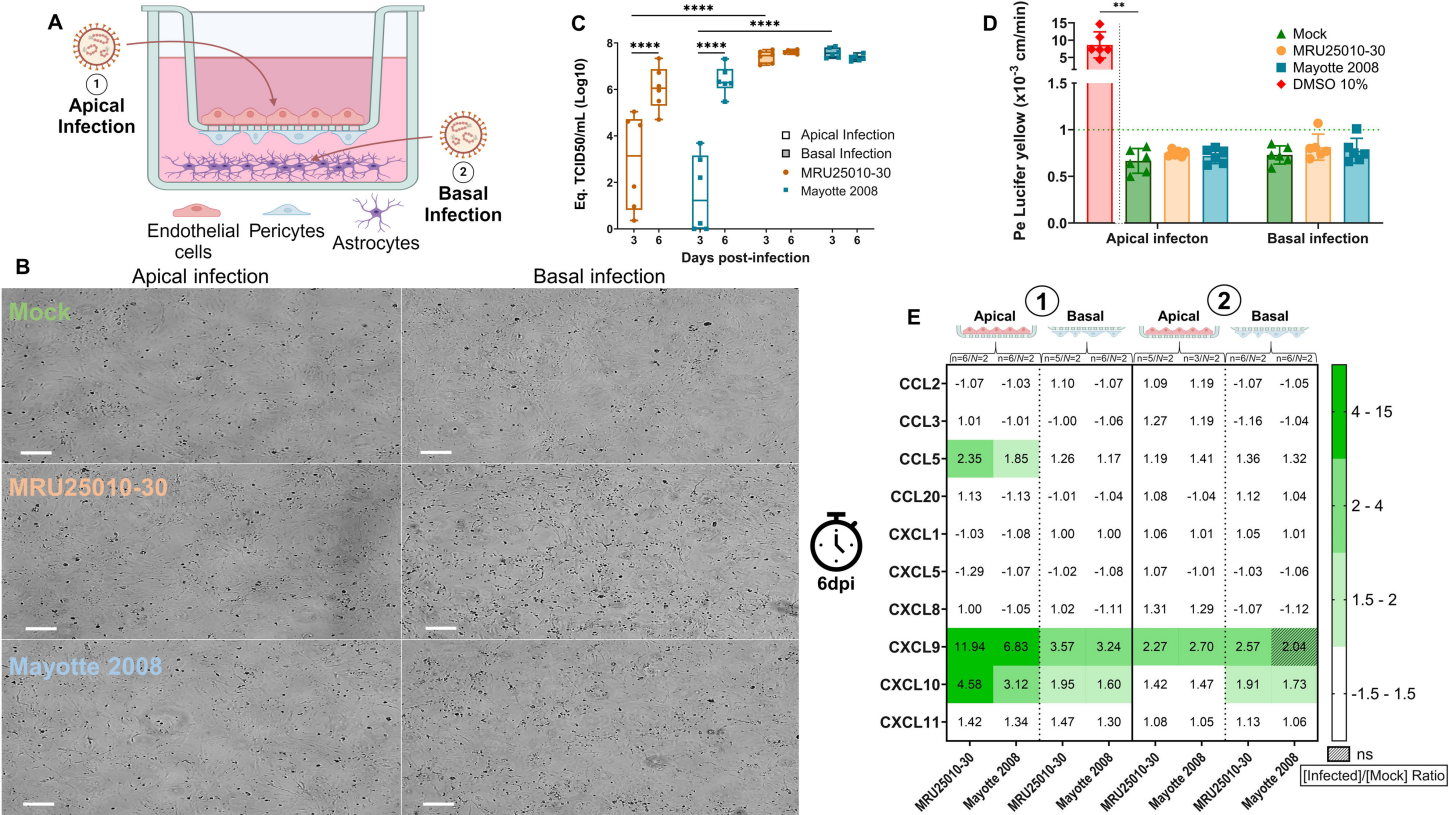
Infection of the blood–brain barrier (BBB) by RVFV led to infection of astrocytes but without disruption of BBB integrity. (A) In order to explore the influence of astrocytes on BBB maintenance during RVFV infection, we used a triculture model: hBBB model (i.e., hBLEC and pericytes) and human astrocytes on the bottom of the well. Infection was performed on the apical side, MOI 1 (1) or on the basal side, MOI 0.1 (2). (B) At 6 days post-infection (dpi) with two different RVFV strains (MRU25010-30 and Mayotte 2008) on the apical side (left) or on the basal side (right), images of astrocytes were acquired by phase-contrast microscopy. Scale bar, 100 µm. (C) From total extracted RNA of RVFV-infected astrocytes after infection of BBB triculture (3 or 6 dpi) on the apical side (clear) or on the basal side (dark), viral titers expressed as the geometric mean of equivalent TCID50/mL (Eq. TCID50/mL) were determined by RTqPCR (generalized linear model, *n* = 6/*N* = 2, ****P < 0.0001). (D) At 6 dpi, the permeability coefficient (Pe) was measured using the Lucifer Yellow transport assay for mock and RVFV-infected BBB triculture. Results are expressed as mean ± 95% confidence interval (CI), and Pe <1 × 10^−3^ cm/min (green dashed line) corresponds to an impermeable/tight BBB. Treatment with 10% DMSO for 30 min corresponds to the positive control of this assay (impaired integrity, Mann–Whitney test, *n* = 6/*N* = 2, **P < 0.01). Generalized linear model test significance between pe of BBB infected by each strain compared with the mock control at different time points (*n* = 6/*N* = 2). (E) Cytokines secreted in RVFV-infected BBB triculture supernatant at 6 dpi at apical infection (1, MOI of 1) or basal infection (2, MOI of 0.1) by two distinct RVFV strains (MRU25010-30 and Mayotte 2008) were measured using FACS multiplex assays and compared with the mock concentration. Results are expressed as protein levels with a ratio of mean protein concentration in infected conditions to that in the mock condition (upregulation in green). Modifications of protein expression represented on the heatmap have a *P* < 0.05 or are hatched when non-significant (for each protein, one-way ANOVA or Kruskal–Wallis on protein concentration values for each compartment independently).

Regulation of BBB integrity is mediated *in vivo* by a complex interplay of NVU cells, in particular by human astrocytes, major CNS glial cells that have already been described as permissive to RVFV infection *in vitro* (47). To explore whether RVFV crossing the hBBB can infect astrocytes and whether the infection can then lead to hBBB disruption, we used an *in vitro* triculture hBBB model with the same culture conditions and viral infection as the previous coculture hBBB model. (Fig. 7A) (41, 57). After 6 days of co-culture, hBBB transwells were transferred on 12-well plates containing human primary astrocytes. To explore whether RVFV can cross hBBB and infect human astrocytes in the compartment at the bottom of the 12-well plates, we performed apical infection of hBLEC at MOI of 1 with RVFV strains MRU25010-30 and Mayotte 2008. Additionally, to directly explore whether RVFV infection of astrocytes can lead to hBBB disruption, we performed basal infection of astrocytes at MOI of 0.1. As expected, basal infection of astrocytes led to a stronger infection of these cells: at 6 dpi and compared with mock and apical infections of hBLEC, MRU25010-30 and Mayotte 2008 appeared to induce a stronger cell toxicity (Fig. 7B). Compared with other conditions, only basal RVFV infection at 6 dpi induced cell shrinkage and rounding. This observation was confirmed by quantifying viral RNA by RT-qPCR in infected astrocytes (Fig. 7C), when the amount of viral RNA was indeed significantly higher in astrocytes directly infected in the basal compartment at 3 dpi. Moreover, the peak of infection (approximatively 10^7^ Eq. TCID50/mL) was reached at 3 dpi for astrocytes infected directly in the basal compartment, and there was no significant difference in viral RNA quantification in the two strains between 3 and 6 dpi. However, following apical infection of hBLEC, infected astrocytes in hBBB triculture showed a significant increase in viral quantification in both strains between 3 and 6 dpi: MRU25010-30 increased from 10^2.9^ to 10^6.1^ Eq. TCID50/mL and Mayotte 2008 increased from 10^1.2^ to 10^6.4^ Eq. TCID50/mL. This delay at 3 dpi between the two routes of infection suggests that RVFV reached astrocytes later when infection is carried out on the apical side of hBLEC. At 6 dpi, there was no longer any significant difference between viral quantification of the two strains or between the two types of infection. These results suggest that infection of astrocytes occurred after infection and crossing of the hBBB by RVFV. Analysis of hBBB integrity by calculating the Pe coefficient showed that despite apical infection of hBLEC or direct basal infection of astrocytes, there was no disruption of RVFV-infected triculture hBBB compared with mock-infected triculture hBBB (Fig. 7C). To explore whether inflammatory signals in hBBB triculture can be modulated by the presence of astrocytes, we quantified several cytokines implicated in the inflammatory response at the protein level at 6 dpi in each strain and with each type of infection (Fig. 7E). Following the apical infection of hBLEC with both RVFV strains, we observed upregulation of three inflammatory cytokines, *i.e*., CCL5, CXCL9, and CXCL10, in the apical compartment. In contrast to the astrocyte-free hBBB culture, we also observed moderate upregulation of CXCL9 and CXCL10 in the basal compartment at 2 dpi. In comparison, when astrocytes were directly infected in the basal compartment, we observed weaker upregulation of the CCL5, CXCL9, and CXCL10 cytokines. CXCL10 was upregulated in the basal compartment in both strains, but only MRU25010-30 induced the upregulation of CXCL9 expression. There was only weak upregulation of CXCL9 in the apical compartment, and no longer any upregulation of CXCL10 and CCL5. These results suggest that only direct infection of hBLEC can induce moderate hBBB inflammation. Moreover, it seems that infection of astrocytes alone did not lead to hBBB inflammation on the apical side by secretion of immune mediators.

Together, these results show that potent infection of astrocytes occured after viral crossing of the hBBB, but suggest that astrocyte infection did not lead to the secretion of immune mediators that could trigger hBBB disruption or inflammation. Thus, inflammation of hBBB seems to be induced only by direct infection of hBLEC by RVFV.

## DISCUSSION

For the first time to our knowledge, our results show that RVFV is able to cross the human BBB *in vitro* by direct infection in a NSs-independent manner but following strain-dependent kinetics. Crossing led to the infection of pericytes and astrocytes in the compartment that mimics brain parenchyma. However, in our model, infection of hBLEC, pericytes or astrocytes did not disrupt BBB, possibly due to gradual elimination of infected cells from the endothelial monolayer with potential maintenance of TJ scaffolds. Our work also highlighted the restriction of hBBB-induced innate immune response by NSs in the apical compartment, thereby potentially favoring RVFV replication.

Interestingly, our work showed that NVU cell types had different permissiveness profiles: hEC and hBLEC are highly permissive, allowing more potent RVFV replication than pericytes. Moreover, only hBLEC triggered an immune response against RVFV, whereas the immune response of pericytes was not at all or only poorly elicited. Surprisingly, pericytes are described in the literature as brain cells mediating inflammation in the CNS by secreting several cytokines, including CCL5 or CXCL10, and it will now be interesting to determine which viral factor inhibits pericyte immune response during RVFV infection (58). Moreover, it appeared that pericytes are more resistant than hBLEC to RVFV infection, and it will now also be interesting to explore which cell factors influence this resistance (cell receptor, cellular machinery, and basement membrane). These cellular factors are probably not correlated with the immune response since pericytes are able to resist without establishment of an immune response, suggesting that resistance to RVFV infection is intrinsic.

We observed no damage to hBLEC monolayers following RVFV infection; however, we only undertook these observations at relatively early time points post-infection. Rupture of the human BBB could be a later event, as it has been reported for human neuropathology, and should consequently be explored (17). Nevertheless, our description of the absence of early BBB breakdown in our *in vitro* model seems to be in agreement with reported observations. Indeed, neurovascular leakage and cerebral hemorrhages are rarely described in humans manifesting RVFV neurological forms and are mainly related to hepatic failure (4, 16, 21), suggesting that the neurovascular system is not strongly impaired during RVFV infection. In addition, RVFV infection of hBLEC did not lead to secretion of factors that favor BBB disruption upon RVFV infection, such as MMPs or inflammatory cytokines (IL-1β, IL6, and TNFα) as it has been observed with other neurotropic viruses, such as the West Nile virus (WNV) (39, 51, 59). Moreover, we observed upregulation of the IFN-λ response during RVFV infection, a type III interferon response mainly upregulated by the epithelium or endothelium, and described to be able to limit viral replication and tighten the BBB (55). In addition, RVFV-infected cells were eliminated from the cell monolayer without disruption of TJ scaffolds, suggesting that natural mechanisms of BBB cell renewal would allow this elimination without disruption of the barrier function. Indeed, it has been reported that apoptotic bovine BMECs continue to express ZO-1 during apoptosis, but BMEC renewal is rarely described in the literature (60). In comparison, the elimination of intestinal epithelial cells during cell renewal, known as cell extrusion, is a much better described mechanism: extruding cells maintain cell–cell contact with surrounding cells by remodeling TJ scaffolds, such as ZO-1, to keep sealing the paracellular space and thus maintain barrier integrity during this process (61). Our results seem to identify a similar mechanism for RVFV-infected hBLEC extrusion from hBBB and it would be interesting to explore this mechanism further in the context of other viral infections.

In addition, BBB function *in vivo* is regulated by a large and complex cell organization. It will be necessary to explore and mimic the NVU more precisely to see whether some types of cells can induce BBB breakdown during RVFV neuroinfection. Here, we showed that RVFV infection of astrocytes did not cause BBB disruption. However, our model did not allow us to mimic the complete interaction between astrocytes and BBB, *i.e.*, protein signals and physical connection between BBB and the end-foot of astrocytes (37, 62, 63). It would be interesting to use a model that allows this connection in order to evaluate if astrocyte mortality, already described during RVFV infection, induces disruption of this connection and thus disruption of BBB integrity (47). However, our results at this stage suggest that RVFV can replicate in the CNS by infection of glial cells without disrupting hBBB, at least at early stages, which is consistent with descriptions of delayed-onset encephalitis in human cases during RVFV infection.

Our investigation showed the establishment of a moderate innate immune response only by hBLEC after RVFV infection, which is polarized to the apical or blood compartment. Interestingly, and despite their described role in immune modulation, astrocyte infection did not lead to an inflammation of hBBB. It is possible that the absence of hBBB inflammation is related to the inhibition of astrocyte innate immune response by the viral protein NSs, as previously reported in the literature (46, 47). Similarly, the RVFV-induced innate immune response in hBLEC is limited by NSs, since infection by the NSs-deleted strain Clone 13 triggered a more potent response. Furthermore, only Clone 13 induced the attachment of lymphocytes at the hBBB at 2 dpi. Indeed, we demonstrated higher upregulation of the chemokines CCL5 and CXCL10 in Clone 13-infected hBLEC supernatants, which are described as factors involved in lymphocyte recruitment at the BBB (39, 64). It has been reported in rodents and in human post-mortem analyses that severe encephalitis was associated with neuro-vasculitis, lymphocytic perivascular cuffing, lymphocytic infiltration on the CNS as well as in the CSF (16, 20–22, 29). We showed that the lack of NSs induces earlier and higher upregulation of innate immune response, although it is possible that in the case of virulent strains, the recruitment of lymphocyte at the BBB occurs at a later stage of infection. Nevertheless, it seems that a strong or dysregulated innate immune response against RVFV at the hBBB is responsible for this recruitment and can enhance neuroinflammation, corroborating the description of severe cases when immune response is dysregulated, and notably upregulation of pro-inflammatory chemokines such as CXCL10 (28, 65).

Interestingly, we found strain-dependent variation in neuroinvasion. Indeed, the strain MRU25010-30, which has already been described as being more virulent than Mayotte 2008 (47), replicated earlier and at higher rates in hBLEC, leading to an earlier release from the basolateral side of the hBBB. However, we showed that the NSs-deleted Clone 13 strain can replicate at the same level as MRU25010-30. These results suggest that the variation in neuroinvasion by the different viral strains is not mediated by the NSs virulence factor, but by other RVFV factors. Moreover, we showed that hEC did not display this strain variability, suggesting that variability can also be mediated by cell factors or characteristics acquired by hBLEC after differentiation by co-incubation with pericytes. These results suggest that the susceptibility of the neurovascular system to RVFV infection can differ from that of peripheral blood vessels, corroborating triggering of hemorrhagic syndrome in peripheral vascular system but not in the human CNS (4, 17). In addition, it would be interesting to identify the virulence factors that drive the ability of RVFV to cross the hBBB and potentially induce neurological disorders. Indeed, during epidemics, it would be important to predict and prevent severe human encephalitis by assessing neuroinvasion ability based on the strain sequences, as is the case for other arboviruses such as *Flaviviridae* whose genetic mutations on the envelope glycoprotein can affect neurotropism (66). In this context, the virulent strain MRU25010-30 was reported to induce severe and even fatal encephalitis in humans during the Mauritanian epidemic in 2010, whereas no encephalitis was reported in Mayotte in 2008 (67, 68).

We demonstrated the potential ability of RVFV to reach the CNS by directly infecting the BBB, but given the complex cellular organisation of the CNS, it seems important to confirm this conclusion by using more complex cellular models, such as assembloids or through post-mortem histological analysis (69). Histopathological analysis of brains of patients who died of RVFV encephalitis are rare, meaning the effective replication of RVFV in the human brain is still uncertain. Nevertheless, such replication was confirmed in NHPs that displayed a neurological form similar to humans (23). It will be interesting to see if RVFV can use other routes to reach human CNS as several arboviruses have several neuroinvasion mechanisms, including the West Nile virus (WNV) that reaches the CNS not only by direct infection, but also *via* the Trojan horse mechanism, by transcytosis across the BBB and also by axonal transport (40, 51). In mouse models, RVFV has been reported to be able to infect neurons, suggesting it can use axonal transport (24). Interestingly, the main route of transmission of RVFV to humans is through aerosolization of viral particles, and it will be interesting to see if the human cribriform plate is also a possible entry route of RVFV into the CNS (27). Concerning the Trojan horse mechanism, it has been reported that dendritic cells are the main target of RVFV during the early stage of infection and could act as Trojan horse as it is reported for other arboviruses (33, 39, 40, 70).

Taken together, our data highlight potential mechanisms of interaction between the CNS and RVFV in humans. Indeed, we have shown that RVFV can reach the human brain by crossing the BBB during the period of viremia, thereby potentially triggering neurological disorder through glial cells, such as astrocytes (47). We highlighted strain variability, suggesting that several factors may influence the ability of RVFV to reach the brain and cause neurological disorders, which is consistent with the inter-individual and inter-epidemic variability in fatality cases and neurological prevalence. Thus, it is vital to explore both the host and viral factors that lead to severe encephalitis in humans to improve public health prevention and treatment during RVFV epidemics.

## MATERIALS AND METHODS

### Virus stock and quantification

Independent stocks of two RVFV virulent strains isolated in the field, strains MRU25010-30 (P1, Mauritania, 2010, KM210508) (71) and Mayotte 2008/00099 (P1, Mayotte, 2008, HE687302-HE687304) (67, 72), as well as the naturally NSs-deleted strain Clone 13 (P3, Bangui) (56), a kind gift from Dr M. Bouloy (*Institut* Pasteur, Paris), were produced using African green monkey kidney Vero cells (ATCC). Virus-containing medium was harvested when the cytopathic effect exceeded 75% and used as virus stock. If the strain is not specified, and only RVFV is mentioned, the two virulent strains were used independently, and the results of the assays were merged to break free from strain-dependent variability. For each virus stock and each experiment, viral titers expressed in TCID50/mL from infected and mock supernatant were determined using the limiting dilution TCID50 method (tissue culture infectious dose, 50%) on Vero cells and the Spearman−Kärber method for the calculations (73). When indicated, viral titers were also determined by RVFV-specific one-step RTqPCR using the AgPath-ID One Step RTqPCR kit (Applied Biosystems, USA) on a LightCycler 96 (Roche, Switzerland) (Tables S1 and S2). When measured by RT-qPCR, viral titers are expressed in equivalent TCID50/mL (Eq. TCID50/mL) based on the standard curve established between Ct values and TCID50/mL values (Fig. S1).

### Cell monoculture and RVFV infection

African green monkey kidney Vero cells were grown in MEM supplemented with 2 mM L-glutamine (Minimal Essential Medium, Gibco, USA) and 10% decomplemented fetal bovine serum (FBS) (Corning, USA) (Passage (*P*) 24–49). Human immortalized brain pericytes provided by Yamaguchi University Graduate School of Medicine (Japan) were maintained in flasks or on plates coated with 0.2% gelatin from porcine skin (Sigma, USA) in DMEM High-Glucose (Dulbecco Minimal Essential Medium, PAN-Biotech, Germany) complemented with 10% FBS and 1% penicillin–streptomycin (P12) (74). Briefly, pericytes were isolated from a patient who had suddenly died from a heart attack by using a cloning cap on dissociated cerebral cortex (74). Thereafter, cells were immortalized *via* sequential transduction with retrovirus-incorporated tsA58 and hTERT genes as described (74). Brain pericyte phenotype of these cells was performed by studying specific markers (α-smooth muscle actin, PDGFR-β, and desmin) (57). CD34^+^ human umbilical blood cord-derived endothelial cells (hECs) were isolated from umbilical cord blood, and purified as described previously (75). Briefly, cells were isolated by Ficoll density gradient and then positively selected twice using the mini-MACS immunomagnetic separation system (Miltenyi Biotec, Bergisch Gladbach, Germany) according to the manufacturer’s recommendations. Cells were subsequently cultured in EGM-2 (Lonza) supplemented with 20% (v/v) fetal bovine serum and 50 ng/mL of VEGF165 (PeproTech Inc.), on 1% gelatin-coated plates. After 15–20 days, hECs were observed in the culture dish. Morphological observations and positive endothelial marker detection (vWF, KDR, CD31) were used to confirm the endothelial phenotype of the cells (hECs). The hECs were then cultured in flasks coated with 0.2% gelatin from porcine skin in endothelial cell growth medium MV2 (Promocell, Germany) supplemented with 0.5% gentamycin. Commercial human primary astrocytes were isolated from human brain and cryopreserved at passage one after confirmation by immunostaining of GAPDH (ScienCell, HA, 1800, lot 25672, USA). Astrocytes were maintained in flasks or on plates coated with 0.01% Poly-D-Lysine (Gibco, USA) according to the manufacturer’s instructions (P3). All cells were grown at 37°C–5% CO_2_. For RVFV infection and depending on the MOI, inocula were prepared by diluting viruses from viral stocks in the appropriate culture medium and were added to the cells for a period of 90 min. A mock control corresponding to the inoculum only-containing medium was included for each infection. At the indicated time point post-infection, supernatants and cells were harvested and stored at −80°C.

### Human BBB and BBB triculture model

The human BBB model consists to co-cultivate the hEC obtained previously with immortalized human brain pericytes for 6 days on transwells (Costar, 0.4 µm, USA) at 37 °C–5% CO_2_ (45). Cells were grown in endothelial cell growth medium MV2 (Promocell, Germany) with hEC on the upper side of the transwell coated with Matrigel (0.2 mg/mL, Corning, USA) and pericytes on the bottom of the transwell coated with 0.2% gelatin. Co-incubation leads to differentiation of hEC into human brain-like endothelial cells (hBLECs) and mimics the main features of human brain microvasculature endothelial cells (hBMECs) of the BBB (41–43). During differentiation, the medium was changed every 2 days. Lucifer Yellow assay (50 µM; Life Technologies, USA) was used to confirm hEC differentiation into hBLEC and to test endothelial permeability (Pe) by measuring and calculating passive diffusion of Lucifer Yellow molecule from the apical to the basal side as described in the literature using the EnSpire Multimode Plate Reader (PerkinElmer, USA, 432/538 nm) (41, 42, 45). The BBB was considered tight/impermeable if Pe ≤to 1 × 10^−3^ cm/min and disrupted if Pe >1 × 10^−3^ cm/min (43, 54). Our BBB triculture model was adapted from the triculture model set up by Artois University (France): astrocytes were plated in 12-well plates in endothelial cell growth medium MV2, and *in vitro* differentiated human BBB model was added to the wells containing astrocytes (57). In the BBB and BBB triculture models, infection was performed as described above only if Pe were ≤1 × 10^−3^ cm/min, and if not specified, inocula were added on the apical side of transwells.

### Indirect immunofluorescence assays

Monolayers of cells were infected on 12-well plates containing coverslips with appropriate coating in the case of monoculture or were infected on the top side of the transwell. Cells were fixed using a 4% paraformaldehyde (PFA, Sigma-Aldrich, USA) solution for 20 min at room temperature (RT) and then stored at 4°C. Cells were permeabilized for 5 min with Triton X-100 0.1% (Sigma-Aldrich, USA) and saturated for 30 min in PBS 1× (Sigma-Aldrich, USA), 10% FBS (Sigma-Aldrich, USA) and 2% BSA (Sigma-Aldrich, USA) before being labeled with primary antibodies for 90 min at RT and then by secondary antibodies for 1 h at RT (Table 1). Labeled coverslips or membranes of transwells were mounted on glass slides with mounting medium (Fluoroshield, Sigma-Aldrich, USA), imaged by inversed confocal ZEISS LSM 900 (Zeiss, Germany) and analyzed with Image J Software bundled with 64-bit Java 1.8.0_322. Infected cells were quantified by counting ≥500 cells per sample on aleatory panoramas of fields acquired with tile modules. When relevant, quantitative cell parameters (volume, Z-axis height) were determined using anti-GAPDH staining, and RVFV intensity ratios were calculated by dividing the mean of RVFV fluorescence intensity of each cell by its volume. Quantitative values were obtained from confocal microscopy images using Imaris Software 10.0.0 (62757) with Imaris cells module 10.0.

**TABLE 1:** Antibodies and dilution used in immunofluorescence assay

Target	First labeling	Dilution (reference)	Secondary labeling	Dilution (reference)
RVFV N structural protein	Monoclonal mouse IgG1 anti-N RVFV	1:50 (Cirad, 15G6-4B8)	Polyclonal donkey IgG anti-mouse IgG 555	1:500 (Invitrogen A31570)
Junction protein ZO-1	Polyclonal rabbit IgG anti-ZO-1	1:50 (Invitrogen, 61–7300)	Polyclonal donkey IgG anti-rabbit IgG 647	1:500 (Invitrogen A31573)
PDGFRβ	Monoclonal rabbit IgG anti-human PDGFRα/β	1:100 (Abcam, Ab32570)	Polyclonal donkey IgG anti-rabbit IgG 647	1:500 (Invitrogen A31573)
GAPDH	Polyclonal goat IgG anti-human GAPDH	1:25 (Abcam, AB9483)	Polyclonal donkey IgG anti-goat IgG 488	1:500 (Invitrogen A-11055)
Actin			ActinGreen (Ready Probes, Alexa488)	1:50 (Thermofischer, 37110)
Nucleus			DAPI	1:1,000 (Sigma-Aldrich, MBD0015)

### Gene expression analysis

To explore the immune response induced by RVFV in hBLEC and pericytes, the total RNA from cell layers was extracted according to the manufacturer’s instructions (RNeasy mini kit, Qiagen, USA) and treated for genomic DNA elimination using RNase-free DNase Set (Qiagen, USA). The quality and the concentration of the total RNA extracted from each sample were checked using the NanoDrop 2000 (Thermo Fisher Scientific, USA). Expression of genes involved in the immune response was then screened by a two-step RT-qPCR (RevertAid First Strand cDNA synthesis, ThermoScientific/LightCycler 480 SYBR Green I Master, Roche) with specific primers for each gene using 400 ng of total RNA on LightCycler 480 equipment (Roche, Switzerland) (Tables S3 and S4). Ct values of targeted genes were normalized using the housekeeping gene *HPRT1*. Fold-change values of transcripts compared with those of uninfected condition were determined using the published 2^-ΔΔCt^ method (76). If fold-change value X was <1, X was converted into −1/X.

### Cytokine and chemokine quantification

To explore the immune response induced by RVFV in hBLEC and pericytes at the protein level, harvested supernatants were tested using two types of LEGENDPlex kits (Biolegend, USA) according to the manufacturer’s instructions. Fluorescent signals (APC, PE) were measured with flow cytometer Canto II (BD Biosciences, USA), and data were analyzed using the online Biolegend software (LEGENDplexTM Data Analysis). For proteins whose concentration was above limit of detection for at least one condition, any modification of protein expression levels is expressed as the ratio of mean protein concentration in infected conditions to that in mock conditions. If protein ratio X was under 1, the value was expressed after −1/X conversion. Upregulation or downregulation of protein expression compared with mock conditions was arbitrarily considered to be above 1.5 or under −1.5, respectively, and confirmed statistically (47).

### LDH assay

To evaluate the cytotoxicity of the infection on hBLEC, LDH activity in apical supernatant of RVFV- or mock-infected hBBB was analyzed at different time points with the LDH CyQUANT Kit (ThermoFischer, USA) on Multiskan MS spectrophotometer (Thermo Labsystems, USA, 492 nm/620 nm) according to the manufacturer’s instructions with slight modifications. Briefly, the same replicates were measured at different time points by harvesting 50 µL of apical supernatant, and maximum LDH activity was measured with independent samples at each time point after incubation with lysis buffer. Spontaneous LDH activity of hBLEC was determined by calculating the mean of the mock-infected sample per experiment, and percentage of cytotoxicity was determined according to the manufacturer’s equation:


$$\%\;Cytotoxicity=\;\left(\frac{Sample\;LDH\;activity-Spontaneous\;LDH\;activity\;}{Maximum\;LDH\;activity-Spontaneous\;LDH\;activity}\right)\times 100$$

### Lymphocyte adhesion assay

Human primary cells were isolated from the buffy coats of healthy donors obtained from the Etablissement Français du Sang. Peripheral blood mononuclear cells (PBMCs) were isolated by density centrifugation using Lymphoprep medium (STEMCELL Technologies, Canada). Primary cells were then cultured in Roswell Park Memorial Institute (RPMI) complemented with 10% FBS and 1% penicillin–streptomycin at 37 °C–5% CO_2_. Non-adherent peripheral blood lymphocytes were collected and grown in culture medium supplemented with 1 µg/mL phytohemagglutinin (PHA, Thermo Fischer Scientific, USA) for 24 h, and then in culture medium supplemented with 10 ng/mL rhIL-2 (ImmunoTools, Germany) for 1 week. T cell isolation was estimated by specific surface staining achieved with antibodies anti-CD3 (UCHT1), anti-CD4 (OKT4), anti-CD14 (HCD14) (Biolegend, USA), and anti-CD8 (REA734, Miltenyi Biotec, Germany). Stained cells were analyzed with LSR Fortessa flow cytometer (BD Biosciences, USA) (Fig. S2). T cells were then co-incubated overnight in harvested supernatant of 2 dpi RVFV- or mock-infected BBB. After co-incubation, T cells were labeled with carboxyfluorescein succinimidyl ester (CFSE) according to the manufacturer’s instructions (CellTrace CFSE Proliferation Kit, 492/517 nm, Invitrogen, USA) and then added on corresponding RVFV- or mock-infected hBLEC for 45 min, as described in the literature (51). After binding, the cells were washed with PBS 1×, and an indirect immunofluorescence assay was performed as described above. After labeling, lymphocytes were counted based on a minimum of two random panoramas of 20 fields per sample.

### Statistical analysis

Each infection was performed in triplicate for each condition. Each experiment corresponds to at least two independent infections. Depending on data distribution (Shapiro test) and on the type of assay, Model (GLM, LM), ANOVAs/Kruskal–Wallis, Student’s t test/Mann–Whitney test, or Spearman/Pearson correlation (*P < 0.05, **P < 0.01, ***P < 0.001, ****P < 0.0001) were used to analyze data with GraphPad Prism 10.2.3 (403) software or RStudio 2021.09.0 (351) (Package lme4 1.1–32, emmeans 1.10.00). When relevant, the sample sizes (n) and the number of independent experiments (*N*) are indicated in the figure and the statistical methods are named in the figure legends.

## Data Availability

All data concerning RNA expression assay are available in Tables S3 and S4.

## SUPPLEMENTAL MATERIAL

Supplemental material jvi.01267-24-s0001.docx Tables S1 to S4; Figures S1 and S2.

ASM does not own the copyrights to Supplemental Material that may be linked to, or accessed through, an article. The authors have granted ASM a non-exclusive, world-wide license to publish the Supplemental Material files. Please contact the corresponding author directly for reuse.
