# Supplementary material for: Rift Valley fever virus is able to cross the human blood–brain barrier in vitro by direct infection with no deleterious effects
Source: J Virol. 2024 Sep 30;98(10):e01267-24. doi: 10.1128/jvi.01267-24 (PMC11494904; doi:10.1128/jvi.01267-24)
Supplement: Supplemental material — Tables S1 to S4; Figures S1 and S2. [file jvi.01267-24-s0001.docx]

| **Target gene** | **Forward primer 5’–3’** | **Reverse primer 5’-3’** | **Probe** | **Reference** |
| --- | --- | --- | --- | --- |
| β-Actin Exon 4-5 | CAGCACAATGAAGATCAAGATCATC | CGGACTCATCGTACTCCTGCTT | TCGCTGTCCACCTTCCAGCAGATGT | [1] |
| RVFV, L (Large) Segment | TGAAAATTCCTGAGACACATGG | ACTTCCTTGCATCATCTGATG | CAATGTAAGGGGCCTGTGTGGACTTGTG | [2] |

**Table S1:** Primers and probes used for RVFV viral quantification by RTqPCR

[1] Toussaint et al., 2007, Bluetongue virus detection by two real-time RT-qPCRs targeting two different genomic segments. J. Virol. Methods 140, 115–123

[2] Bird et al., 2007, Highly sensitive and broadly reactive quantitative reverse transcription-PCR assay for high-throughput detection of Rift Valley fever virus. J. Clin. Microbiol. 45, 3506–3513

**Table S2:** Cycling conditions used for RVFV viral quantification by RTqPCR

| **Reference of the kit used** | **Cycling conditions** | | | |
| --- | --- | --- | --- | --- |
| AgPath-ID One Step RTqPCR (Applied Biosystems, USA) | cDNA synthesis | Inactivation | Amplification (40 cycles) | |
|  | 45°C, 10min | 95°C, 10min | 95°C, 15s | 60°C, 1min |


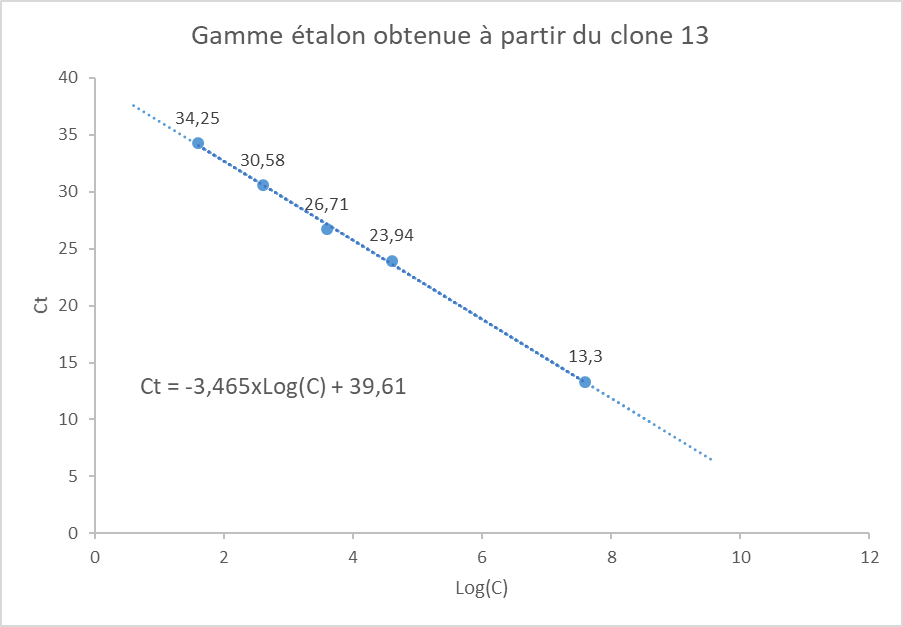


**Figure S1:** Standard curve between Ct values of Clone 13 measured by RTqPCR on Vero cells and viral titers expressed in TCID50/mL Log10 (Log (C)).

**Table S3:** Primers used for mRNA expression analysis by RTqPCR

| **Target gene** | **Full Name/Accession number** | **Forward primer 5’–3’** | **Reverse primer 5’-3’** | **Reference** |
| --- | --- | --- | --- | --- |
| *CASP3* | Caspase 3 / NM_004346 | TGGCGAAATTCAAAGGATG | TAACCCGGGTAAGAATGTGC | [1] |
| *CASP9* | Caspase 9 / NM_001229 | GCAGTAACCCCGAGCCAGATG | CCGGAGGAAATTAAAGCAACCAG | [1] |
| *CCL5* | C-C motif ligand 5 / NM_002985 | CTCATTGCTACTGCCCTCTGCGCTCCTGC | GCTCATCTCCAAAGAGTTGATGTACTC | [2] |
| *CLDN5* | Claudin 5 / NM_001130861.1 | TTAACAGACGGAATGAAGTT | AAGCGAAATCCTCAGTCT | [3] |
| *CSF2* | Colony stimulating factor 2 / M11220.1 | ATGTTTGACCTCCAGGAGCC | GGTGATAATCTGGGTTGCACA | [4] |
| *CXCL10* | C-X-C motif ligand 10 / NM_001565 | TATTCCTGCAAGCCAATTTTGTC | TCTTGATCGCCTTCGATTCTG | [5] |
| *CXCL11* | C-X-C motif ligand 11 / NM_005409 | GACGCTGTCTTTGCATAGGC | GGATTTAGGCATCGTTGTCCTTT | [6] |
| *HPRT1* | hypoxanthine phosphoribosyltransferase 1 / NM_000194.3 | AGCTTGCTGGTGAAAAGGAC | TTATAGTCAAGGGCATATCC | [7] |
| *IFNA1* | Interferon alpha 1 / NM_024013 | TGGCTGTGAAGAAATACTTCCG | TGTTTTCATGTTGGACCAGATG | [8] |
| *IFNA2* | Interferon alpha 2 / NM_000605 | CTTGAAGGACAGACATGACTTTGGA | GGATGGTTTCAGCCTTTTGGA | [9] |
| *IFNB1* | Interferon beta 1 / NM_000605 | GTCTCCTCCAAATTGCTCTC | ACAGGAGCTTCTGACACTGA | [10] |
| *IFNG* | Interferon gamma / NM_000619 | CTTTAAAGATGACCAGAGCATCCA | ATCTCGTTTCTTTTTGTTGCTATTGA | [11] |
| *IFNL1* | Interferon lambda 1 / NM_172140.2 | CACGCGAGACCTCAAATATGTG | AGGGTGGGTTGACGTTCTCA | [12] |
| *IFNL2/3* | Interferon lambda 2/3 / NM_172138.2/NM_001346937.2 | GCCACATAGCCCAGTTCAAGTC | GGCATCTTTGGCCCTCTTAAA | [12] |
| *IL1B* | Interleukin 1B / NM_000576 | ATGATGGCTTATTACAGTGGCAA | GTCGGAGATTCGTAGCTGGA | [13] |
| *IL6* | Interleukin 6 / NM_000600 | CCAGGAGCCCAGCTATGAAC | CCCAGGGAGAAGGCAACTG | [14] |
| *ISG15* | Interferon stimulated gene 15 / NM_005101 | TGTCGGTGTCAGAGCTGAAG | GCCCTTGTTATTCCTCACCA | [15] |
| *MMP2* | Matrix Metalloproteinase 2 / NM_004530 | GAGCTCTATGGGGCCTCTCC | CGTCACAGTCCGCCAAATGA | [16] |
| *MMP9* | Matrix Metalloproteinase 9 / NM_004994.3 | GGGAAGATGCTGCTGTTCA | AACTCACTCCGGGAACTCAC | [17] |
| *OCLN* | Occludin / NM_002538.4 | TTCTGGATCTCTATATGGTTCA | CCACAACACAGTAGTGATAC | [3] |
| *TGFB1* | Tumor Growth Factor beta 1 / NM_000660.7 | AAGGACCTCGGCTGGAAGTG | CCCGGGTTATGCTGGTTGTA | [18] |
| *TNFA* | Tumor Necroting factor alpha / NM_000594 | CAGCCTCTTCTCCTTCCTGAT | GCCAGAGGGCTGATTAGAGA | [19] |
| *TJP1* | Tight Junction Protein 1 / NM_003257.5 | CCTGAACCAGTATCTGATAA | AATCTTCTCACTCCTTCT | [3] |

[1] Li Z, Guo D, Yin X, Ding S, Shen M, Zhang R, Wang Y, Xu R. Zinc oxide nanoparticles induce human multiple myeloma cell death via reactive oxygen species and Cyt-C/Apaf-1/Caspase-9/Caspase-3 signaling pathway in vitro. Biomed Pharmacother. 2020 Feb;122:109712. doi: 10.1016/j.biopha.2019.109712. Epub 2019 Dec 30. PMID: 31918281.

[2] Vaday GG, Peehl DM, Kadam PA, Lawrence DM. Expression of CCL5 (RANTES) and CCR5 in prostate cancer. Prostate. 2006 Feb 1;66(2):124-34. doi: 10.1002/pros.20306. PMID: 16161154.

[3] Chang KF, Liu CY, Huang YC, Hsiao CY, Tsai NM. Downregulation of VEGFR2 signaling by cedrol abrogates VEGF‑driven angiogenesis and proliferation of glioblastoma cells through AKT/P70S6K and MAPK/ERK1/2 pathways. Oncol Lett. 2023 Jun 22;26(2):342. doi: 10.3892/ol.2023.13928. PMID: 37427338; PMCID: PMC10326829.

[4] Gilliland G, Perrin S, Blanchard K, Bunn HF. Analysis of cytokine mRNA and DNA: detection and quantitation by competitive polymerase chain reaction. Proc Natl Acad Sci U S A. 1990 Apr;87(7):2725-9. doi: 10.1073/pnas.87.7.2725. PMID: 2181447; PMCID: PMC53763.

[5] Eichholz K, Mennechet FJ, Kremer EJ. Human coagulation factor X-adenovirus type 5 complexes poorly stimulate an innate immune response in human mononuclear phagocytes. J Virol. 2015 Mar;89(5):2884-91. doi: 10.1128/JVI.03576-14. Epub 2014 Dec 24. PMID: 25540380; PMCID: PMC4325717.

[6] Gao YJ, Liu L, Li S, Yuan GF, Li L, Zhu HY, Cao GY. Down-regulation of CXCL11 inhibits colorectal cancer cell growth and epithelial-mesenchymal transition. Onco Targets Ther. 2018 Oct 23;11:7333-7343. doi: 10.2147/OTT.S167872. PMID: 30425523; PMCID: PMC6205823.

[7] Siednienko J, Nowak J, Moynagh PN, Gorczyca WA. Nitric oxide affects IL-6 expression in human peripheral blood mononuclear cells involving cGMP-dependent modulation of NF-κB activity. Cytokine. 2011 Jun;54(3):282-8. doi: 10.1016/j.cyto.2011.02.015. Epub 2011 Mar 16. PMID: 21414799.

[8] Zhao Q, Liang D, Sun R, Jia B, Xia T, Xiao H, Lan K. Kaposi's sarcoma-associated herpesvirus-encoded replication and transcription activator impairs innate immunity via ubiquitin-mediated degradation of myeloid differentiation factor 88. J Virol. 2015 Jan;89(1):415-27. doi: 10.1128/JVI.02591-14. Epub 2014 Oct 15. PMID: 25320320; PMCID: PMC4301122.

[9] Löseke S, Grage-Griebenow E, Wagner A, Gehlhar K, Bufe A. Differential expression of IFN-alpha subtypes in human PBMC: evaluation of novel real-time PCR assays. J Immunol Methods. 2003 May 1;276(1-2):207-22. doi: 10.1016/s0022-1759(03)00072-3. PMID: 12738374.

[10] Faure E, Poissy J, Goffard A, Fournier C, Kipnis E, Titecat M, Bortolotti P, Martinez L, Dubucquoi S, Dessein R, Gosset P, Mathieu D, Guery B. Distinct immune response in two MERS-CoV-infected patients: can we go from bench to bedside? PLoS One. 2014 Feb 14;9(2):e88716. doi: 10.1371/journal.pone.0088716. PMID: 24551142; PMCID: PMC3925152.

[11] Mallampalli RK, Adair J, Elhance A, Farkas D, Chafin L, Long ME, De M, Mora AL, Rojas M, Peters V, Bednash JS, Tsai M, Londino JD. Interferon Lambda Signaling in Macrophages Is Necessary for the Antiviral Response to Influenza. Front Immunol. 2021 Nov 25;12:735576. doi: 10.3389/fimmu.2021.735576. PMID: 34899695; PMCID: PMC8655102.

[12] Mihm S, Frese M, Meier V, Wietzke-Braun P, Scharf JG, Bartenschlager R, Ramadori G. Interferon type I gene expression in chronic hepatitis C. Lab Invest. 2004 Sep;84(9):1148-59. doi: 10.1038/labinvest.3700135. PMID: 15208644. [13] Wang H., et al. STAT3 Regulates the Type I IFN-mediated antiviral response by interfering with the nuclear entry of STAT1. Int J Mol Sci. 2019;20.

[14] Chen Y, Pawlikowska L, Yao JS, Shen F, Zhai W, Achrol AS, Lawton MT, Kwok PY, Yang GY, Young WL. Interleukin-6 involvement in brain arteriovenous malformations. Ann Neurol. 2006 Jan;59(1):72-80. doi: 10.1002/ana.20697. PMID: 16278864.

[15] Bektas N, Noetzel E, Veeck J, Press MF, Kristiansen G, Naami A, Hartmann A, Dimmler A, Beckmann MW, Knüchel R, Fasching PA, Dahl E. The ubiquitin-like molecule interferon-stimulated gene 15 (ISG15) is a potential prognostic marker in human breast cancer. Breast Cancer Res. 2008;10(4):R58. doi: 10.1186/bcr2117. Epub 2008 Jul 15. PMID: 18627608; PMCID: PMC2575531.

[16] Sumiya R, Terayama M, Hagiwara T, Nakata K, Sekihara K, Nagasaka S, Miyazaki H, Igari T, Yamada K, Kawamura YI. Loss of GSTO2 contributes to cell growth and mitochondria function via the p38 signaling in lung squamous cell carcinoma. Cancer Sci. 2022 Jan;113(1):195-204. doi: 10.1111/cas.15189. Epub 2021 Nov 21. PMID: 34726807; PMCID: PMC8748250.

[17] Constant O, Maarifi G, Barthelemy J, Martin MF, Tinto B, Savini G, Van de Perre P, Nisole S, Simonin Y, Salinas S. Differential effects of Usutu and West Nile viruses on neuroinflammation, immune cell recruitment and blood-brain barrier integrity. Emerg Microbes Infect. 2023 Dec;12(1):2156815. doi: 10.1080/22221751.2022.2156815. PMID: 36495563; PMCID: PMC9815434.

[18] Abdalla AO, Kiaii S, Hansson L, Rossmann ED, Jeddi-Tehrani M, Shokri F, Osterborg A, Mellstedt H, Rabbani H. Kinetics of cytokine gene expression in human CD4+ and CD8+ T-lymphocyte subsets using quantitative real-time PCR. Scand J Immunol. 2003 Dec;58(6):601-6. doi: 10.1111/j.1365-3083.2003.01348.x. PMID: 14636415.

[19] Li Z, Chao TC, Chang KY, Lin N, Patil VS, Shimizu C, Head SR, Burns JC, Rana TM. The long noncoding RNA THRIL regulates TNFα expression through its interaction with hnRNPL. Proc Natl Acad Sci U S A. 2014 Jan 21;111(3):1002-7. doi: 10.1073/pnas.1313768111. Epub 2013 Dec 26. PMID: 24371310; PMCID: PMC3903238.

**Table S4:** Cycling conditions used for mRNA expression analysis by RTqPCR

| **Reference of the kit used** | **Cycling conditions** | | | | | |
| --- | --- | --- | --- | --- | --- | --- |
|  | Preincubation | cDNA synthesis | Amplification (40 cycles) | | | Melting curve/Inactivation |
| RevertAid First Strand cDNA synthesis (Fischer Scientific, USA) | 65°C, 5min | 42°C, 60min |  | | | 70°C, 5min |
| LightCycler 480 SYBR Green I Master (Roche, Switzerland) | 95°C, 10min |  | 95°C, 15s | 60°C, 15s | 72°C, 25s | 95°C, 5s then 65°C to 97°C |


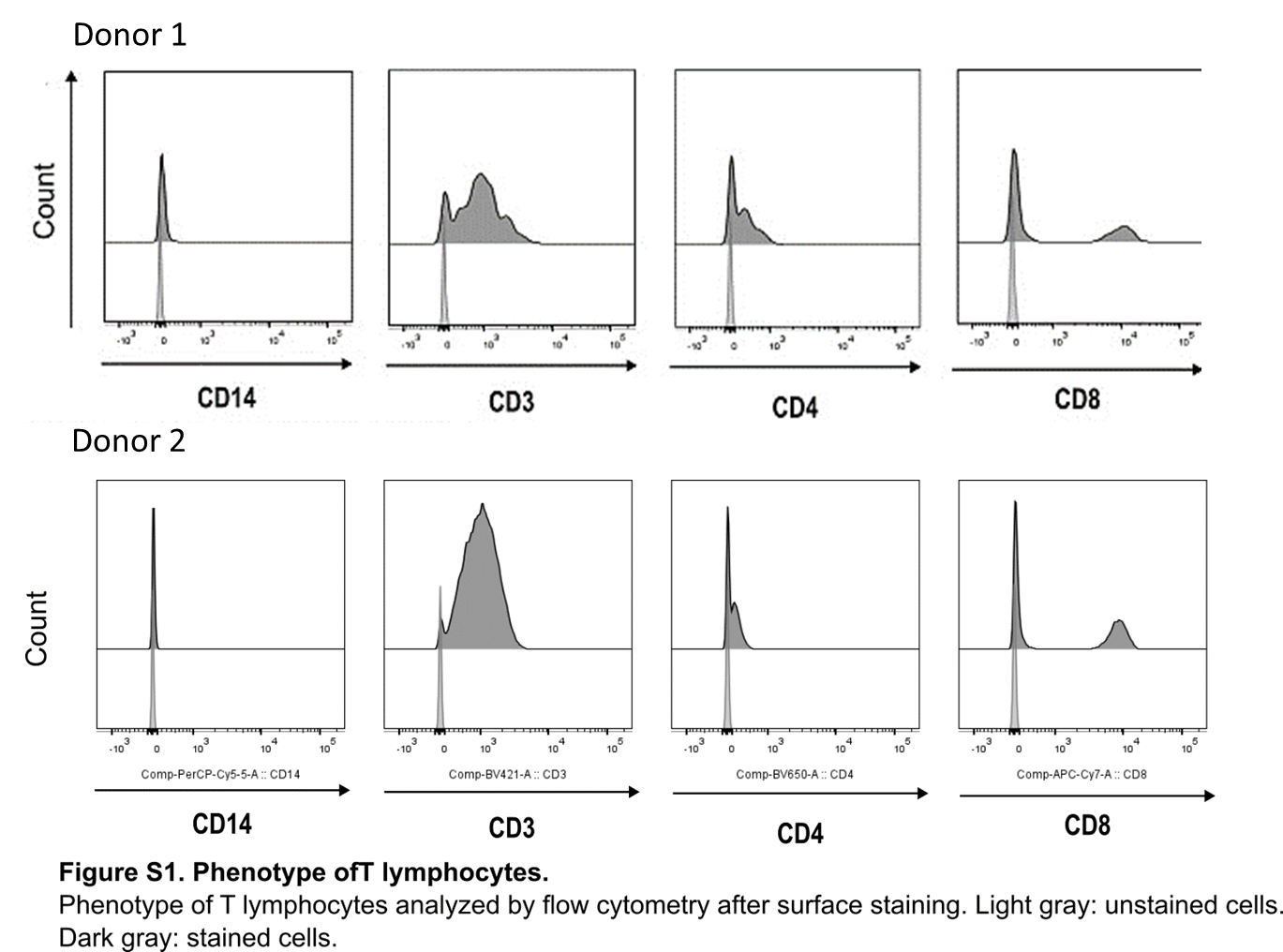


**Figure S2:** Phenotype of T cells analyzed by flow cytometry after surface staining (CD14, CD3, CD4 and CD8) for cell isolated from donor 1 (top panels) and donor 2 (bottom panels). Light gray: unstained cells. Dark grey: stained cells
